# Comprehensive lipid structure annotation via photochemical epoxidation and mass spectrometry

**DOI:** 10.1007/s00216-025-05953-6

**Published:** 2025-06-20

**Authors:** Jing Yu, Belal Alshaar, Sven Heiles

**Affiliations:** 1 Leibniz-Institut für Analytische Wissenschaften – ISAS - e.V., Otto-Hahn-Straße 6B, 44227 Dortmund, Germany; 2 Lipidomics, Faculty of Chemistry, University of Duisburg-Essen, Universitätsstrasse 5, 45141 Essen, Germany

**Keywords:** Mass spectrometry, Lipid derivatization, Tandem mass spectrometry, Isomers, Epoxidation

## Abstract

Cells tightly regulate lipid structures to fulfill cellular functions and to respond to external stimuli. The biochemical details of the processes that determine lipid structure alterations are often not fully understood. In this manuscript, we present a new epoxidation strategy of unsaturated lipids, which allows the annotation of lipid head group, fatty acid composition, C = C bond position, C = C bond geometry, and *sn*-isomerism when derivatized lipids are analyzed with corresponding separation and mass spectrometry methods. Tandem mass spectra of epoxidized deprotonated or protonated lipids provide information on head groups, fatty acids, and C = C positions, while MSⁿ of alkali metal adducts reveals *sn*-isomer compositions. Separation of epoxidation products via reversed-phase liquid chromatography (RPLC) not only distinguishes lipid C = C position and *sn*-isomers, remaining non-reacted unsaturated lipid C = C bonds photo-isomerize to reveal C = C *E*/*Z* configurations. To demonstrate the capabilities of the methodology, C = C positions of LPEs, LPCs, TGs, DGs, PCs, PSs, and PEs are annotated for bovine heart and liver extracts in RPLC-MS^2^ experiments, and shotgun MS^n^ is employed to characterize 56 PC *sn*-isomers in HeLa and H9c2 cell lines.

**Graphical Abstract:**

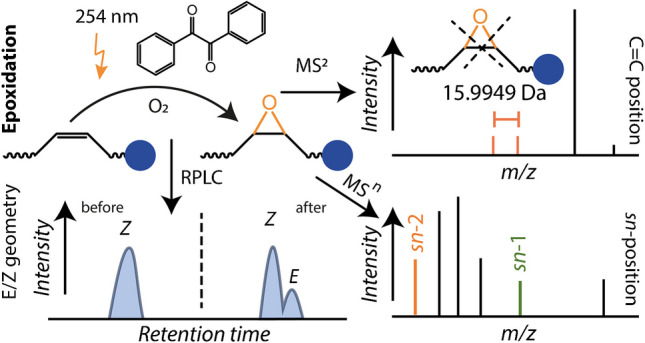

**Supplementary Information:**

The online version contains supplementary material available at 10.1007/s00216-025-05953-6.

## Introduction

A plethora of lipids exists in living organisms that perform various functions ranging from cell membrane formation to energy storage and signaling [1, 2]. To rationalize lipid functions and characterize the dynamic adaptation of cellular lipid structures to external stimuli, the field of lipidomics strives to detect as many lipids as possible and decipher their molecular architecture in one sample [3]. This endeavor, however, is a demanding bioanalytical task as subtle changes in the molecular makeup of lipids often hinder unambiguous annotation of lipid structures, and reliable predictions of lipid isomers are complicated by the intertwined nature of enzymatic processes involved in lipid metabolism. For example, cells adapt their phospholipid fatty acid (FA) composition to their needs in a biochemical cascade termed Lands cycle [4]. In this cycle, the attachment of FAs to lysolipids occurs with the help of lysophospholipid acyltransferase enzymes. Whereas the involved enzymes in the Lands cycle are mostly known, their specificity and selectivity have not been studied in detail. Zhao et al. recently showed that upregulation of phosphatidylcholine (PC) 18:1/16:0 in tumor tissue is indeed connected with the increased lysophosphatidyl acyltransferase-1 (LPCAT1) expression compared to surrounding healthy material, showcasing the need for structurally resolved lipidomics workflows [5].

For this reason, multiple new methods have been developed that track lipid structure changes, mainly relying on mass spectrometry (MS) [3]. Sensitive detection with direct infusion methods, or shotgun MS, of lipids has been implemented on multiple state-of-the-art mass spectrometric platforms. By electrospray ionization (ESI), detection of hundreds of lipids is routinely feasible by making use of high mass resolution and high mass accuracy capabilities of modern MS instruments [6]. To resolve the structural complexity of lipid mixtures, hyphenation of MS methods with separation systems such as liquid chromatography (LC) [7] or ion mobility spectrometry (IMS) [8] can facilitate disentangling complex samples. LC–MS and shotgun MS lipid annotations additionally make use of tandem mass spectrometric methods to reveal lipid class and FA composition.

To gain further insights into lipid structures and reveal FA C = C (DB) positions, DB geometries, and stereospecific numbering (*sn*) isomers, specialized derivatization, separation, and/or tandem mass spectrometry methods are required [9–11]. One of the first lipid structure selective methods, termed ozone-induced dissociation (OzID), was introduced by Blanksby and co-workers utilizing ozone to cleave DB bonds [12]. OzID has been used to investigate DB positions as well as *sn*-isomers of lipids. Most recently, it was used in a large-scale shotgun MS study to investigate *sn*-isomer abundances in different cell lines. In this study, Michael et al. revealed that *sn*-isomer compositions can differ between cell lines, and they identified very long chain FAs attached to phospholipids [13]. Structure selective fragments are also obtained by ultraviolet photodissociation (UVPD) [14–16], a method pioneered for lipids by Brodbelt and co-workers. By exciting lipid ions with UV light, DB positions, *sn*-isomers, and cyclopropanation sites are identified. But also, other methods that use laser light can yield information about lipid structures [17–20]. The selection of ions adducted to lipids can regularly alter fragmentation pathways, facilitating the formation of fragments that are indicative of DB or methyl branching sites [21, 22]. Lipid fragmentation can also be influenced by introducing new functional groups into lipids by derivatization methods prior to ionization [9, 23–25]. Whereas a large number of derivatization strategies have been developed, epoxidation [26, 27] and Paternò-Büchi (PB) [28–31] workflows are most regularly used. All these methodologies can provide some additional information for the overall lipid structure, such as DB position, *sn*-isomers, or FA modifications. However, only electron impact excitation of ions from organics has provided direct evidence that *E*/*Z* DB isomers of lipids can be distinguished based on fragment ion abundances [32].

To further improve the annotation capabilities for isomers in lipidomics workflows, IMS and LC have been used in combination with these advanced tandem mass spectrometry methods [9]. For example, OzID has been combined with reversed-phase LC (RPLC) for the analysis of the structural diversity of FAs in human plasma [33]. RPLC was also employed to separate PB mixtures [34] and epoxidized lipids [35, 36]. Whereas analysis of PB reaction products is most often coupled with hydrophilic interaction liquid chromatography [37], analysis of non-derivatized lipids in PB reaction mixtures can be used to identify *E*/*Z* isomers [31] of unsaturated lipids.

Here, we report a new epoxidation workflow that contributes to these ongoing efforts to fully characterize lipid structures in lipidomics studies. The epoxidation method relies on the photochemical reaction of C = C bonds with molecular oxygen photocatalyzed by benzil [38]. The reaction is performed in a flow reactor, resulting in about 80% reaction yield for monounsaturated FA moieties. Whereas the formed epoxides can reveal DB positions as reported before, *sn*-isomer annotation based on MS^n^ experiments of sodiated lipids is generalized for most GPL classes. We furthermore demonstrate that injecting reaction products into RPLC allows improving separation of DB positions and *sn*-isomers after epoxidation compared to non-reacted lipids. Whereas these features are most likely generalizable to all epoxidation strategies, a special trait of the benzil-assisted epoxidation is the *E*/*Z* isomerization of DBs in non-reacted and reacted compounds. This enables *E*/*Z*-geometry annotation in monounsaturated FA moieties or lipids with DBs in only one FA. To demonstrate the capabilities of the method for shotgun and LC–MS lipidomics workflows, we identified DB positions and geometries of PCs, PEs, PSs, DGs, and TGs in complex polar lipid extracts and revealed 56 *sn*-isomers in HeLa and H9c2 cell extracts.

## Materials and methods

### Chemicals and materials

Acetonitrile (ACN), methanol, and water (HiPerSolv CHROMANORM®) were purchased from VWR International GmbH (Darmstadt, Germany). Dichloromethane and isopropanol were purchased from Carl Roth GmbH + Co. KG (Karlsruhe, Germany). Benzil (1,2-diphenylethane-1,2-dione) and methyl *tert*-butyl ether (MTBE) were purchased from Merck (Darmstadt, Germany). Ammonium formate and sodium acetate were obtained from Sigma-Aldrich. Lipid standards, polar bovine liver extract, and polar bovine heart extract were purchased from Avanti Polar Lipids (Alabaster, AL, USA). FA 18:0;9Ep(cis) was obtained from Cayman Chemical (Ann Arbor, MI, USA). Lipid nomenclature is described in the Supplementary Material.

### Sample preparation and cell cultures

The human cervical HeLa S3 (ACC 161, DSMZ, Germany) and rat H9c2 myoblast cells (Sigma Aldrich, Germany, RRID: CVCL_0286) were grown in Dulbecco’s Modified Eagle’s Medium (DMEM, PAN Biotech) with added 10% fetal bovine serum (Sigma Aldrich) as well as 1% 10,000 U/mL penicillin and streptomycin. The medium of incubated cells was removed, and cells were resuspended in Dulbecco’s Phosphate Buffered Saline (DPBS, PAN Biotech). Next, DPBS was removed, and adherent cells were detached with trypsin/EDTA (PAN Biotech) by incubating for 2 min at 37 °C and 5% CO_2_. The cell suspension was diluted with DMEM and spun down (300 g, 5 min). Cell pellets were resuspended in DPBS, counted in a hemocytometer to obtain 10^6^ cell aliquots, and were transferred into 2-mL homogenization tubes. After removing DPBS, cell pellets were snap frozen in liquid nitrogen and kept at − 80 °C till use.

Lipid extracts from cells were prepared using the MTBE extraction method, with minor modifications. Briefly, after adding 375 µL of methanol to 1 million HeLa or H9c2 cells, the mixture was incubated at RT for 1 h while vortexing at 600 rpm. Next, 1.25 mL of MTBE and 313 µL of deionized water were added, followed by centrifugation at 10,000 rpm for 10 min. The upper MTBE phase was carefully collected, dried in a SpeedVac, and stored at − 80 °C for subsequent use.

For preparing working solutions for epoxidation, lipid standards (FAs, PCs, PE, PG, PS, PI, PA) were diluted in ACN to a final concentration of 0.2 mg/mL as stock. Benzil was dissolved in ACN:water (1:1, v/v) and sonicated for 6 min to achieve a final concentration of 2 mg/mL. The working solution was prepared by adding 50 µL benzil stock solution and 425 µL ACN:water (1:1, v/v) to 25 µL of lipid standard stock solution. For liver/heart and cell extracts, 50 µL benzil was added into 15 µL liver/heart extract (25 mg/mL) stock solution and dried cell extract sample, respectively, and ACN:water (1:1, v/v) was added to get to the final volume of 500 µL. The working solution was vortexed for 20 s and stored at 4 °C until further use.

### Photochemical epoxidation

Reported conditions in this section are optimized reaction conditions. More information about the optimization of these parameters is included in the Supplementary Material. For the reaction of standards, a volume of 500 µL working solution was injected in the flow reactor (Figure S1), and the outlet was either connected to a HESI ion source for reaction monitoring or collected in a vial for further processing after observing epoxidized lipids in the mass spectrometer. The injection pump flow rate, controlled by the instrument tune software, was set to a 40 µL/min; the total reaction time is 20.5 min per injection. For reaction monitoring, the outlet of the fluorinated ethylene propylene capillary was fed into a Velos Pro mass spectrometer equipped with a HESI-II probe. For shotgun MS and LC–MS experiments of standards, the reaction solution was collected and used as described in the sections “Mass spectrometry” and the Supporting Information. For extracts, the same reaction protocol was applied, but the reacted solution was collected, dried, and stored at − 80 °C until further analysis. Heart and liver extracts were reconstituted in 100 µL of isopropanol:methanol (1:1, v/v) before LC–MS/MS measurements. Cell extracts were reconstituted using the same solvent system, with the addition of 200 µM sodium acetate aqueous solution for shotgun MSⁿ measurements. The flow reactor was cleaned with 500 µL dichloromethane, 500 µL acetonitrile, and 500 µL water after every reaction to prevent contamination and carryover.

### Mass spectrometry

MS^1^ and MS^n^ measurements of oxidation products were conducted using a Velos Pro or an Eclipse mass spectrometer (Thermo Fisher Scientific, Bremen, Germany). The instruments were equipped with either a heated electrospray ionization (HESI-II) probe (Thermo Fisher Scientific) or a nanoESI source. For reaction monitoring, HESI settings were ± 3.5 kV as spray voltage, an inlet temperature of 300 °C, a sheath gas flow of 10, and an aux gas flow of 8. NanoESI settings were 1 kV for MS^n^ shotgun measurements after loading 15 µL of the reaction solution into pulled nanoESI emitters as described before [28]. The isolation window for all MS^n^ was ± 0.5 *m*/*z*, the collision setting (CID) was 35 NCE, and the MS^n^ resolution was 15,000 at *m*/*z* 400. Detailed information about RPLC and additional MS parameters are provided in the Supplementary Material.

## Results and Discussion

### Benzil-photosensitized epoxidation of lipids

**Fig. 1:**
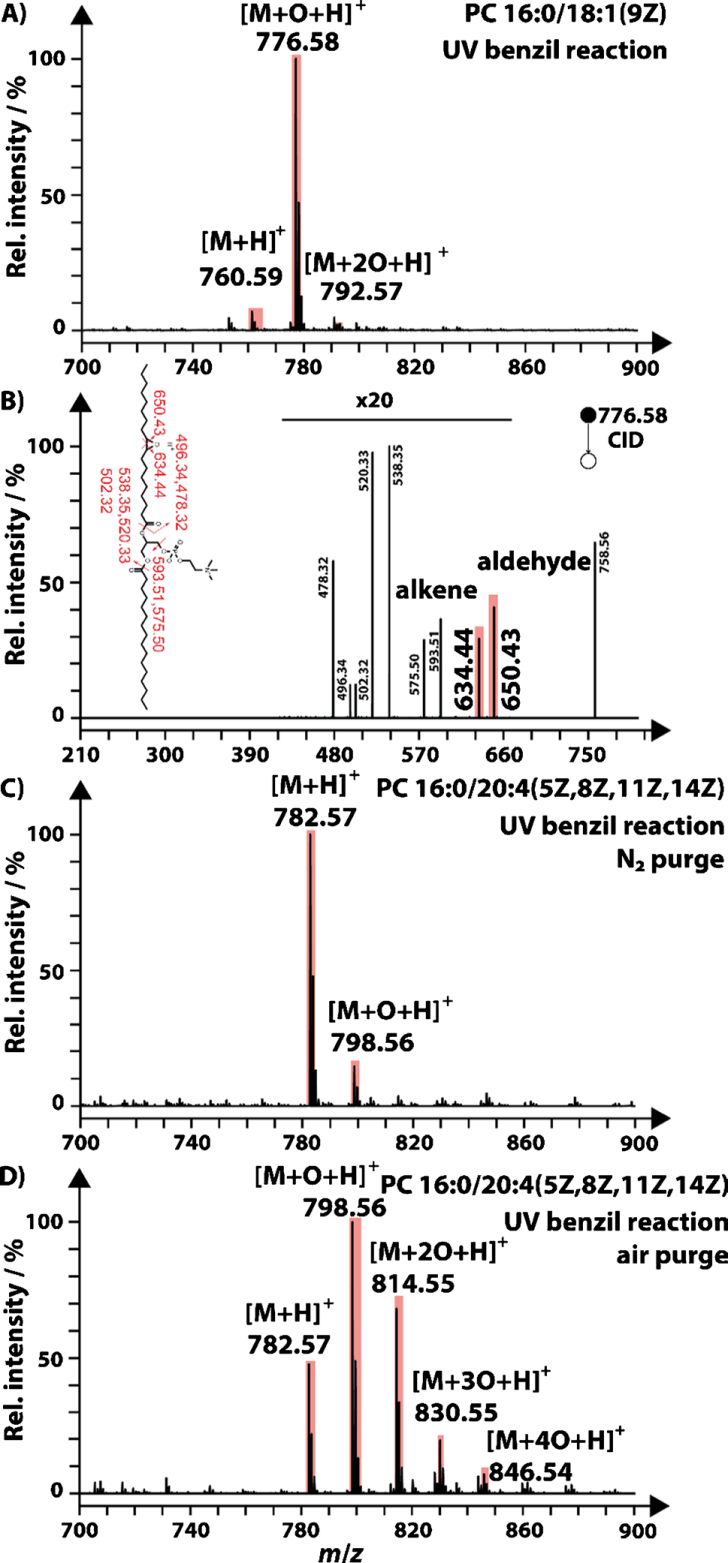
Benzil-induced oxidation of unsaturated lipids via 254 nm light. **A** Mass spectrum of reaction products after oxidation of PC 16:0/18:1(9Z) and **B** MS^2^ of the base peak at *m*/*z* 776.58 with fragments labeled and DB-selective ions highlighted. Mass spectra of reaction products after oxidation of PC 16:0/20:4(5Z,8Z,11Z,14Z) were obtained following 4 h of purging the reaction solution with nitrogen (**C**) and synthetic air (**D**). Some signals are labeled with corresponding *m*/*z* values and highlighted in red

To investigate photochemical lipid derivatization, a flow reactor was designed, illustrated in Figure S1. Multiple potentially photoreactive aldehydes and ketones were tested with the aim to derivatize unsaturated lipids in a 254-nm-triggered PB reaction. Whereas most compounds reported in the literature, for instance, 3-acetylpyridine [28], yielded the desired oxetanes, the mass spectrum of PC 16:0/18:1(9Z) spiked with benzil (Fig. 1A) did not contain corresponding PB products. Instead, a pronounced mass spectrometric signal at *m*/*z* 776.58 was present that was shifted + 15.99 Da relative to the precursor at *m*/*z* 760.59 (Fig. 1A). An additional signal was also observed at + 31.98 Da compared to the protonated precursor *m*/*z* but in lower intensity. These results are consistent with the first signal at *m*/*z* 776.58 originating from epoxidation, ketone formation, or hydroxylation of PC 16:0/18:1(9Z) that is initiated by 254 nm light and requires benzil.

The absence of similar results in experiments with PC 32:0 suggests that DBs are the primary reactive sites (Figure S2). Additionally, the MS^2^-CID spectrum of [PC 16:0/18:1(9Z) + O + H]^+^ resulted in the formation of DB position specific fragments (Fig. 1B), consistent with literature reports for epoxidized PCs [27]. Because we did not observe the addition of benzil to unsaturated lipids or any lipid-benzil-associated fragments and no other oxygen source was added to the reaction solution, we hypothesized that dissolved molecular oxygen could be involved in the reaction. To confirm the photoactivation of molecular oxygen by benzil, we purged the reaction solution with N_2_ for 4 h prior to initiating the photochemical reaction of PC 16:0:/20:4 (5Z, 8Z, 11Z, 14Z). This resulted in the reduction of the + 15.99 Da and 31.98 Da signals by a factor of ~ 20 after UV irradiation in the flow reactor (Fig. 1C) compared to purging the reaction solution with air (Fig. 1D). These results are consistent with the involvement of dissolved O_2_ in the reaction. Our findings are in line with prior reports in the organic chemical literature that showed the photoactivation of O_2_ by benzil [38, 39]. These results indicate that the use of benzil in a water/ACN solution can effectively epoxidize unsaturated lipids.

**Scheme 1:**
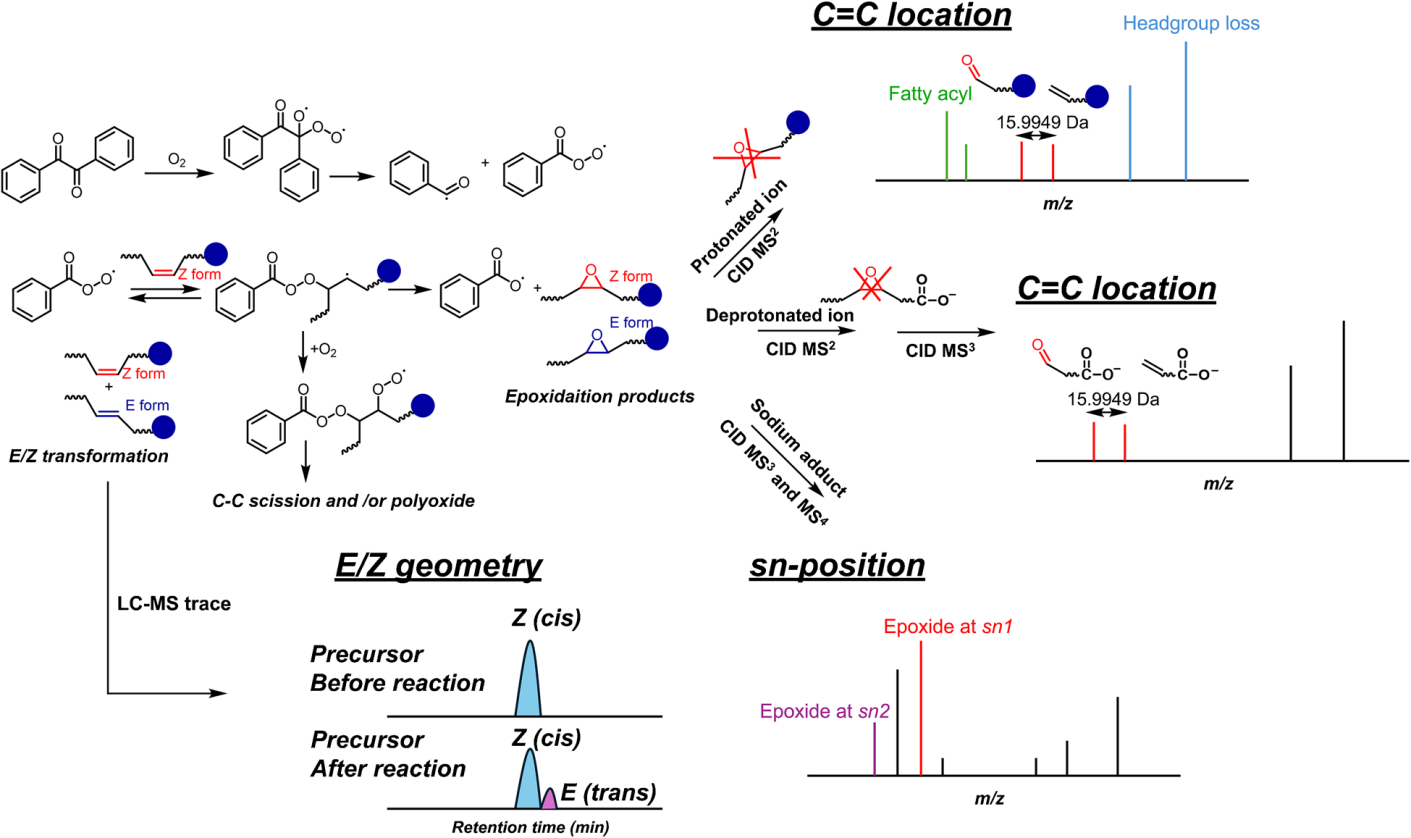
Schematic of the benzil-mediated photo-epoxidation of unsaturated lipids and its practical application in shotgun MS and LC–MS workflows

Consequently, we varied the reaction time, solvent composition, and benzil concentration to optimize the mono-epoxidation yield and minimize byproducts. Results for the method optimization are reported in Figure S3-S7 and Table S4. To be more sensitive to potential side reactions and optimize the mono-epoxidation yield, PC 16:0/20:4 was used for these optimizations. As shown in Figure S3 and indicated in Table S4, a mixture of ACN:water 1:1 minimizes side reaction products during the reaction and guarantees solubility of lipids. A benzil concentration of 0.2 mg/mL gave the highest mono-epoxidation yield and minimal side reactions (Figure S4), and the effect of varying the reaction time by changing the light-accessible surface is shown in Figure S5. As shown in Figure S6, these optimized reaction conditions result in an epoxidation yield above 60% for FAs and glycerolphospholipids (GPLs). The day-to-day and experiment-to-experiment reproducibility of the optimized reaction is less than ± 2.5% even though the ambient temperature and pressure were only stable within ± 1.5 °C and ± 80 mbar, respectively (Figure S7). These combined results evidence that the described photochemical reaction occurs between benzil, molecular oxygen, and DBs in lipids. Based on these observations and literature reports [38, 39], a plausible reaction mechanism is shown in Scheme 1. Scheme 1 also contains analytical applications of the described epoxidation method as detailed in the following sections.

### Fatty acid isomer epoxidation, separation, and DB isomerization

**Fig. 2:**
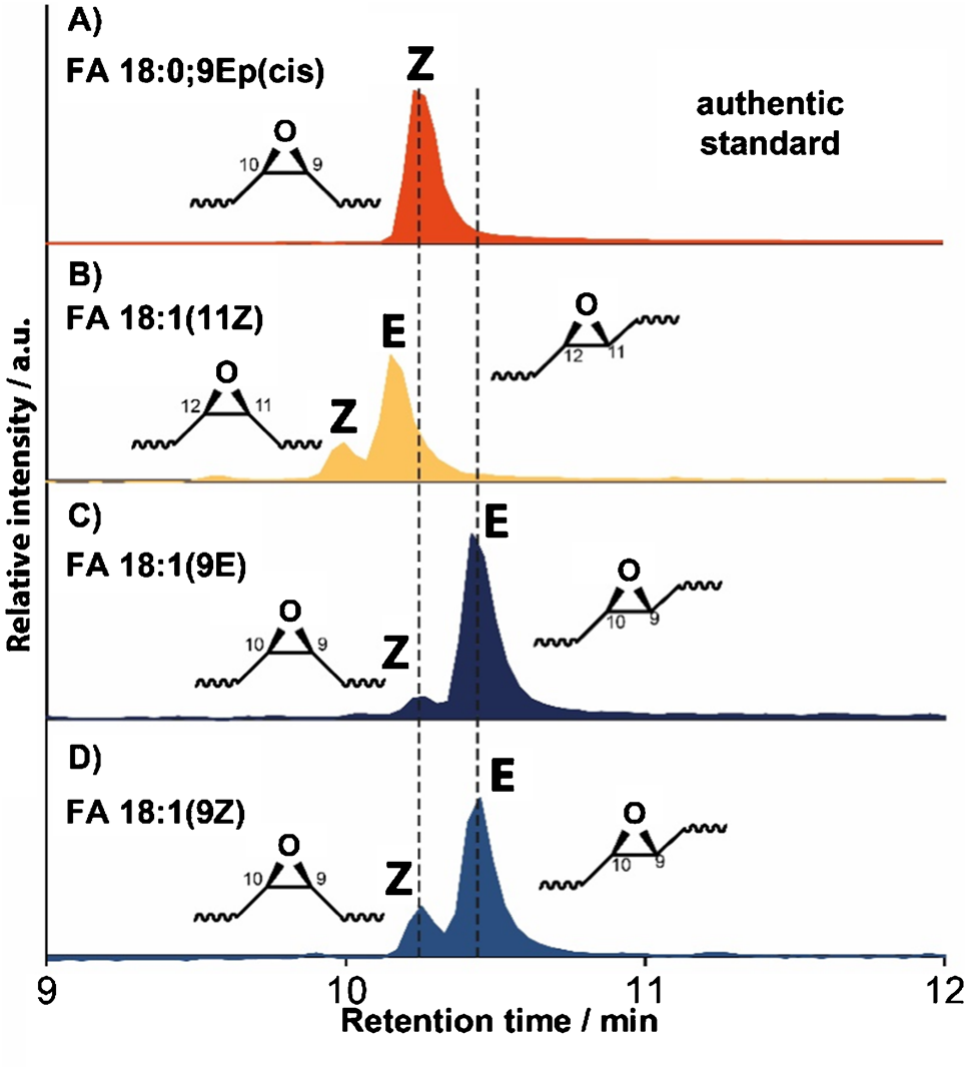
EIC of *m*/*z* 297.24 in negative-ion mode. The EIC of the **A** authentic standard FA 18:0;9Ep(cis) is compared to the benzil-mediated oxidation products of **B** FA 18:1(11Z), **C** FA 18:1(9E), and **D** FA 18:1(9Z). The retention times of FA 18:0;9Ep(cis) and FA 18:0;9Ep(trans) are indicated by dashed lines

Next, we investigated whether the products formed after light-induced benzil oxidation were primarily epoxides or if isobaric reaction products, such as alcohols or ketones, were formed. For this purpose, we conducted negative-ion mode LC–MS/MS measurements of oxidized authentic standards: FA 18:1(9Z), FA 18:1(9E), and FA 18:1(11Z). Their tandem mass spectra and chromatographic traces were compared to those of the FA 18:0;9Ep(cis) standard. The extracted ion chromatograms (EICs) of *m*/*z* 297.24 are shown in Fig. 2, while the MS/MS results are presented in Figure S8. A single chromatographic peak is obtained for FA 18:0;9Ep(cis) (Fig. 2A), together with the MS^2^ results (Figure S8G) indicating the presence of only one isomeric form of this epoxidized compound. Similarly, the authentic standards of FA 18:1 isomers prior to the reaction (Figure S9) do not show two peaks. For the oxidation products of FA 18:1(9E) and FA 18:1(9Z), the first signal at ~ 10.2 min co-elutes with FA 18:0;9Ep(cis). This indicates that photochemical oxidation of FA 18:1(9E) and FA 18:1(9Z) results in a product that contains one additional oxygen atom compared to the precursor, has MS^2^ spectra consistent with the epoxidation of a Δ9 DB (Figure S8A and S8C), and has the same retention time as FA 18:0;9Ep(cis). Therefore, we conclude that this first signal in Fig. 2C and D at 10.2 min results from the formation of FA 18:0;9Ep(cis) after photochemical benzil oxidation. Fragment ions upon CID of the second chromatographic peak at ~ 10.4 min are in line with an epoxide between Δ9 and Δ10, consistent with Δ9 DBs in both FA precursors (Figure S8B and S8D). However, the separation between the first signal at 10.2 min and that at 10.4 min indicates the presence of a second isomeric epoxide. Based on literature results for epoxides [39] and the presence of the epoxide between Δ9 and Δ10, this second chromatographic peak in Fig. 2C and D indicates the formation of FA 18:0;9Ep(trans). As the two separated peaks occur after the oxidation of FA 18:1(9E) and FA 18:1(9Z) and the proposed epoxidation mechanism induced by benzil proceeds via an intermediate without a DB or an oxygen-containing heterocycle, these results are consistent with the isomerization of the precursor DB geometry before the oxirane is formed. This is further supported by the LC–MS result for FA 18:1(11Z) after oxidation in which epoxides are formed at Δ11 position based on the MS^2^ spectra (Figure S8E and S8F), but two chromatographic peaks are present. Comparison of the relative peak area in Fig. 2C and D also indicates that isomerization in both directions, i.e., from *cis* to *trans* and from *trans* to *cis*, is possible, with the *trans* geometry of the resulting epoxide being the major product. Due to the absence of any other major isobaric peaks in Figure S8B and S8D, the combined results indicate that signals that differ from the precursor by multiples of the mass of atomic oxygen are mainly due to the formation of epoxides at the position of the DB bonds in the precursor molecule. Unlike other reported epoxidation strategies, epoxidation via benzil-mediated photosensitization of dissolved molecular oxygen results in the isomerization of the DB bond during the reaction of FAs.

### RPLC of photo-epoxidized glycerophospholipids

**Fig. 3:**
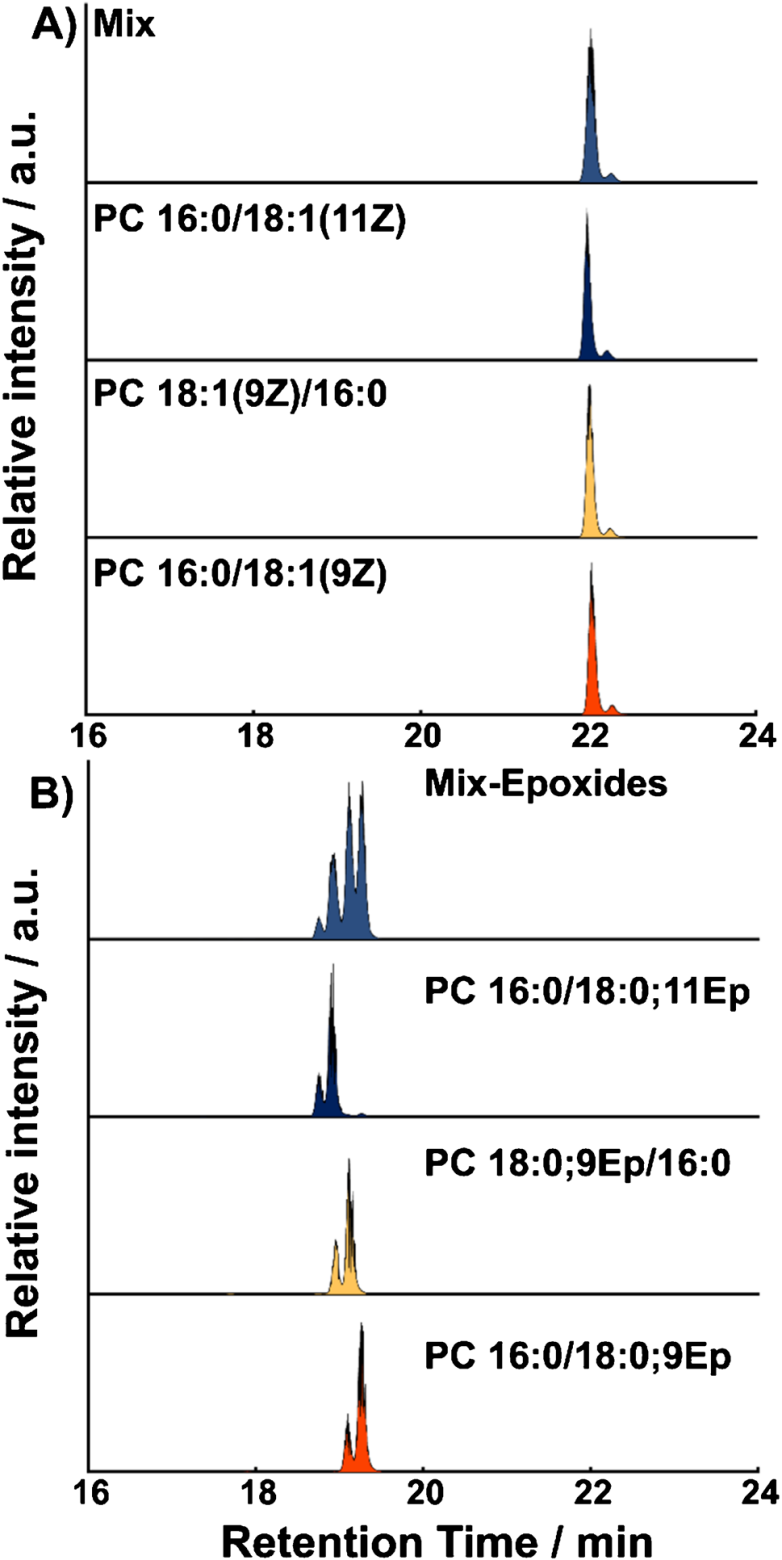
EIC of **A** [PC 34:1 + H]^+^ and **B** [PC 34:0;Ep + H]^+^ isomers and corresponding mixtures. The EIC of authentic analytic standards after photochemical epoxidation is shown for [PC 16:0/18:1(11Z) + H]^+^, (yellow) [PC 18:1(9Z)/16:0 + H]^+^, and (orange) [PC 16:0/18:1(9Z) + H]^+^ in blue, yellow, and orange, respectively. Corresponding epoxidation products have the same color code. Equimolar mixtures of all three reaction mixtures are shown on top in faded blue color

Subsequently, we investigated the impact of the described workflow on the ability to structurally annotate and separate GPL isomers. Structure-selective fragments for DB positions and *sn*-isomers can be obtained by MS^n^ in positive-ion mode as described in Figure S10 and Supplementary Note 3. For example, the MS^2^ of protonated PE 16:0/18:0;9Ep generates diagnostic signals to identify the head group (*m*/*z* 652.38), fatty acyl composition (*m*/*z* 496.30), and the location of the DB (*m*/*z* 451.37 and *m*/*z* 467.37) (Figure S10A and S10B). Additionally, the *sn*-characteristic ions can be obtained by MS^3^ of [M + Na]^+^ ions resulting in a diagnostic feature at *m*/*z* 291.23 for PE 16:0/18:0;9Ep (Figure S10D). Based on the structure-diagnostic ions obtained upon benzil-photoepoxidation, we investigated whether separation with RPLC could further improve structure annotations by isomer separation including *E*/*Z* annotations as discussed for FAs (Fig. 2). Results of individual and mixed lipid isomers are shown in Fig. 3 for three PC 34:1 isomers and Figure S11 for other lipid isomers after benzil-induced photo-epoxidation.

The extracted ion chromatogram (EIC) of non-epoxidized PC 34:1 after the epoxidation reaction reveals two signals that are separated in the mixture of all three isomers but do not significantly differ. The first signal at around 22 min RT (Fig. 3A) overlaps with the PC 34:1 mixture prior to the reaction (Figure S12). The coelution of *sn*- and DB position GPL isomers is also consistent with literature reports that demonstrated the inability to separate most GPL *sn*- and DB position isomers when using a RP column, unless the attached FA residues on *sn*−1 and *sn*−2 significantly differ in length or DB number [40]. This is in contrast to *E*/*Z* DB stereoisomers that are regularly separated [40]. Therefore, the second signal in Fig. 3A at ~ 22.2 min most likely indicates the presence of the *E* DB bond isomer. This is supported by (1) the separation of PC 18:1/18:1 *E*/*Z* isomers and formation of additional signals after photo-epoxidation, with the *E* isomers eluting later than *Z* configurations (Figure S11), and (2) the isomerization and separation of FA *E*/*Z* isomers on the same column, as shown in Fig. 2. This combined evidence allows us to conclude that DBs of non-epoxidized GPLs can isomerize within the reaction mixture enabling subsequent RPLC separation. Similar results are obtained for PEs (Figure S13).

This is also in line with results from Feng et al. that showed that photochemical reactions involving radical processes can lead to *E*/*Z* isomerization facilitating DB geometry determination [31]. For GPL containing monounsaturated FA moieties, photo-epoxidation allows to determine the *E*/*Z* configuration. This is accomplished by determining the RT difference between the lipid precursor prior to and after epoxidation. Due to the *E*/*Z* interconversion (Scheme 1) induced by radical intermediates, additional chromatographic features appear after benzil-induced reaction. If the newly formed signals shift to longer RTs, DBs with *Z* geometry were present prior to the reaction, whereas a shift to shorter RTs after the reaction indicates an *E* → *Z* transformation.

Next, we investigated the ability to separate epoxidized GPLs and obtain corresponding structural information in MS^2^ and MS^3^ experiments. In Fig. 3B, the chromatographic separation of three PC 34:0;Ep isomers and their mixture is shown. Unlike the insufficient isomer separation for the PC 34:1 mixture in Fig. 3A, epoxidation of the DB improves the ability to partially or fully separate *sn*- and DB position isomers (Fig. 3B). PC 16:0/18:0;11Ep (blue) elutes earlier than PC 18:0;9Ep/16:0 (yellow) and PC 18:1;9Ep/16:0 (yellow) earlier than PC 16:0/18:0;9Ep (orange) but all epoxidized compounds exhibit two peaks. Based on the results for FAs (Fig. 2), GPLs without epoxidation (Fig. 3A and Figure S12), and MS^2^/MS^3^ data that confirm the presence of only one DB position and *sn*-isomers (Figure S14), the first peak is most likely due to the *Z* DB forming a *cis*-oxirane, but the majority of the DB isomerizes to form a *trans*-oxirane. Similar results are obtained for PEs (Figure S13) and GPLs with one polyunsaturated FA and one saturated FA (Figure S20). Improved separation of epoxidized GPLs on RP columns is consistent with literature reports by Nakanishi et al. that showed the separation of GPL *sn*-isomers containing polyunsaturated FAs after hydroxylation and Carpanedo et al. after hydroxylation/epoxidation [35].

### Workflows for the benzil-mediated epoxidation of GPLs

The established photochemical epoxidation strategy can be used in multiple bioanalytical scenarios as highlighted in Scheme 1. (A) For shotgun MS, DB positions are available in positive- or negative-ion mode MS^n^ for protonated or deprotonated compounds, respectively (Figure S15 and S16), as also reported for other epoxidation methods [27]. Even though most likely a universal trait of epoxidized GPLs, here, we demonstrated that the addition of sodium salts to benzil-epoxidized lipids yields *sn*-isomer information in MS^n^ experiments for GPL classes (Figure S17). (B) Separation of the reaction solution via RPLC enables improved lipid isomer separation (Fig. 3B) after epoxidation compared to non-reacted compounds prior to assigning DBs or *sn*-isomers as described in A via MS^n^. The chromatographic profile of non-epoxidized lipids in the reaction solution will additionally reveal DB geometries if monounsaturated FAs are linked to GPLs or only one FA moiety contains multiple DBs (Fig. 2, Fig. 3A, and Figure S20) if compared to profiles prior to the reaction.

### Annotating lipid isomers in liver and heart extracts with RPLC-MS

**Fig. 4:**
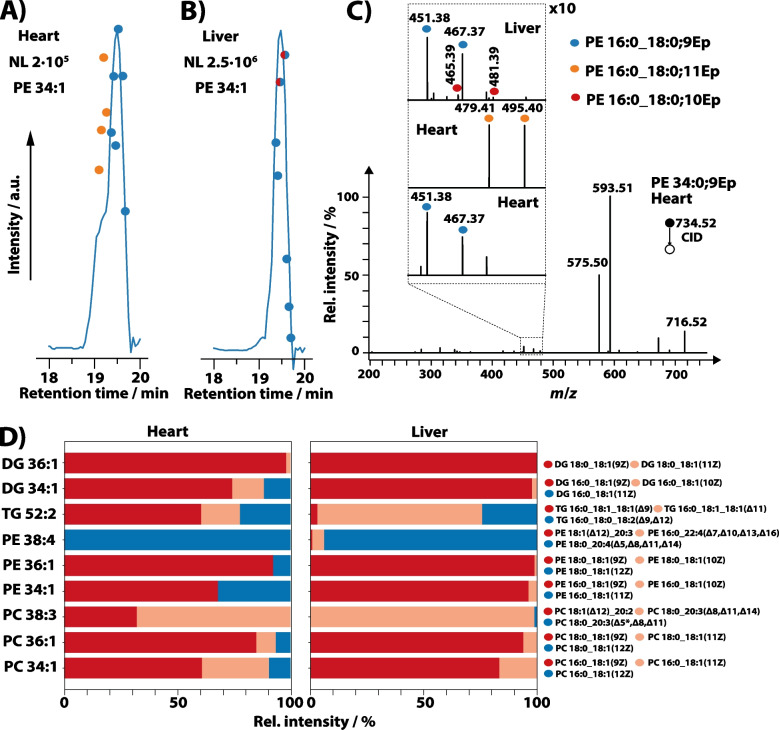
Lipid isomer composition of selected lipids in bovine heart and liver lipid extracts upon photochemical benzil epoxidation. EIC of protonated PE 34:0;Ep in **A** bovine heart and **B** liver extracts. **C** MS^2^ of protonated PE 34:0;Ep in bovine heart and in the inset of heart and liver extracts. DB-selective fragment peaks are highlighted. MS^2^ acquisitions during the LC run are highlighted by colored points in **A** and **B** that correspond to the MS^2^ spectra shown in **C**. Relative DB and FA isomer intensities of some lipids in heart and liver extracts from LC-MS^2^ experiments. Relative errors are ± 5% and asterisk (*) indicates that not all single MS^2^ spectra contain the diagnostic DB position

Next, we used the developed workflow to investigate lipid isomer abundances in bovine liver and heart extracts by RPLC-MS^2^, following the workflow shown in Scheme S1. After performing untargeted lipidomics of the unreacted extracts, we selected a target list of lipids of different classes (PE, PC, PS, LPE, LPC, DG, TG) at the sum composition level to investigate their lipid composition after photochemical benzil-epoxidation. Lipids for which full assignment of head group, FA composition, and DB positions in positive-ion mode as [M + H]^+^ ions after photochemical epoxidation was possible are listed in Table S5 (heart extract). A comparison between the identified lipid composition and their relative isomeric abundances for some of these lipids in liver and heart extracts is shown in Fig. 4, and additional examples are provided in Figure S21.

The comparison between the EIC of PE 34:0;Ep in heart and liver extract is shown in Fig. 4A and B, respectively. Whereas the EIC of PE 34:0;Ep of both extracts exhibits the maximum intensity at 19.5 min, the chromatographic trace of the heart extract is broadened towards shorter RT. The corresponding MS^2^ from 19.5 min is shown in Fig. 4C and the magnification of the *m*/*z* range of 445 to 510 for DB-diagnostic ions is shown in the inset for different RTs and extracts. For RT 19.5 min in heart extract, only fragments at *m*/*z* 451.38 and *m*/*z* 467.37 are observed, consistent with the presence of a *n-9* DB. Because the same spectrum contains information about the FAs (Table S5), the highest intensity feature in the EIC is assigned to PE 16:0_18:0;9Ep. These spectra are highlighted by blue circles in Fig. 4A–C. Additionally, using LC traces of the corresponding non-epoxidized lipid after the reaction, we assign this compound as PE 16:0_18:1(9Z). All investigated monounsaturated lipids for these two extracts contained only *Z* DB geometries as expected (see Figure S22 for examples). The shoulder at 19.2 min in heart extract contains fragment ions at *m*/*z* 479.41 and *m*/*z* 495.40, revealing that PE 16:0_18:0;11Ep is responsible for the broadening of the chromatographic peak (MS^2^; orange circles). For liver extracts, the broadening of the EIC is not present, and the majority of MS^2^ spectra are assigned to PE 16:0/18:0;9Ep, with the exception that two MS^2^ spectra contain low-intensity signals at *m*/*z* 465.39 and *m*/*z* 481.39 (Fig. 4C). These signals are consistent with low levels of PE 16:0_18:0;10Ep. Therefore, these results in combination with LC traces from unreacted lipids after epoxidation reveal that heart extract and liver extract contain PE 16:0_18:1(9Z) as well as PE 16:0_18:1(11Z) and PE 16:0_18:1(9Z) as well as low levels of PE 16:0_18:1(10Z), respectively.

Results for other lipid compositions are shown in Fig. 4D. Because *E*/*Z* assignment was not possible for PUFAs, only DB position and FA composition are reported. Photo-epoxidation of unsaturated lipids and RPLC-MS^2^ enables the assignment of the FA composition as well as DB positions for DG, TG, PC, PE, PS, LPC, and LPE, enabling to differentiate the isomer composition between the two extracts. For example, DG 36:1 in liver contains only DG 18:0_18:1(9Z), whereas the same lipid is composed of DG 18:0_18:1(9Z) and DG 18:0_18:1(11Z) in heart (Fig. 4D). Besides revealing DB positional isomers, FA compositional isomers can be resolved. This is the case for TG 52:2, for which TG 16:0_18:1_18:1(Δ9), TG 16:0_18:1_18:1(Δ11), and TG 16:0_18:0_18:2(Δ9, Δ12) are observed in both extracts, but their relative abundance differs (Fig. 4D). Note that only one DB position is determined for TGs with two FA 18:1 and not the combination of both DB positions. Besides the well-known DB positions for FA 18:1 at Δ9 and Δ11, our method identifies DBs at Δ10 and Δ12 in low relative abundance. These results are consistent with PB assignments of these DB isomers from bovine extracts and were recently also observed in low abundance for FAs [25, 30, 33]. All other DB positions are in line with canonical DB positions and have the highest abundance (above 85%). That the determination of DB positions is not limited to lipids with only a few DBs is showcased for PEs and PCs. For example, PE 38:4 consists only of PE 16:0_22:4(Δ5, Δ8, Δ11, Δ14) in heart extract, but liver is composed of PE 16:0_22:4(Δ5, Δ8, Δ11, Δ14), PE 18:0_20:4(Δ7, Δ10, Δ13, Δ16), and low abundant PE 18:1(Δ12)_20:3 (Fig. 4D). These results demonstrate that the coupling of photo-chemical epoxidation with chromatographic separation enables the assignment of DB positions, FA composition, and for lipids with monounsaturated FA moieties the DB geometry in complex mixtures, supporting RPLC-MS workflows for global lipidomics.

### Charting *sn*-isomers of HeLa and H9c2 cells

**Fig. 5:**
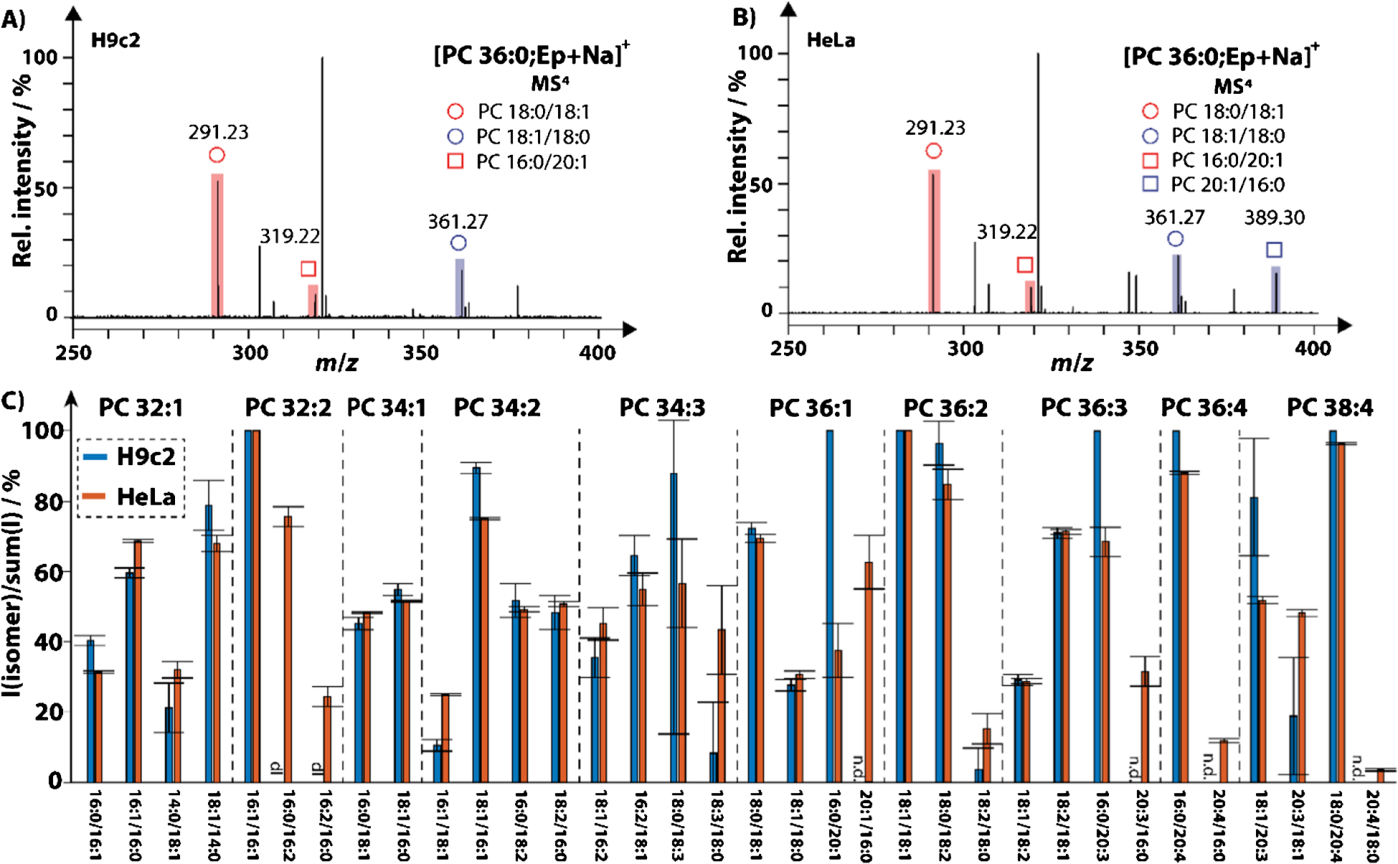
Shotgun MS results for [PC 36:0;Ep + Na]^+^ for **A** H9c2 and **B** HeLa cells revealing *sn*-isomer diagnostic fragment ions. **C** Comparison of the intensity (*I*) of diagnostic fragments of a *sn*-isomer divided by the sum of the intensities of both *sn*-isomers. Mean values and error bars are from three biological replicates, n.d. indicates not detected *sn*-isomers, dl indicates “detected-in-low-abundance,” and bars with only one *sn*-isomer identified do not have error bars

The *sn*-isomer composition of GPLs for different cell lines can differ, and polyunsaturated FA moieties do not necessarily only populate *sn*−2 positions. This highlights the tight level of control cells exert to build individual lipidomes, as recently reported by Michael et al. for four different cell lines [13]. To demonstrate the availability of *sn*-isomer information from photo-epoxidized unsaturated GPLs, we used the developed methodology to chart the *sn*-isomer abundance of PCs in shotgun MS experiments (Fig. 5). For this purpose, HeLa and H9c2 cells were chosen, as they are commonly used models in cancer and cardiovascular disease research, respectively. For example, Fig. 5A and B shows the MS^4^ spectra of sodiated PC 36:0;Ep with highlighted *sn*-isomer diagnostic ions. Whereas the tandem mass spectrometric result for H9c2 cells, rat cardiomyocyte cells, contains signals for the *sn*-isomer pair PC 18:0/18:1 and PC 18:1/18:0, as well as PC 16:0/20:1, fragmentation of epoxidized and sodiated PC 36:1 in the immortalized cervical cancer HeLa cell line yields the same fragments as H9c2 but additionally reveals the presence of PC 20:1/16:0. In addition to the four *sn*-isomers for PC 36:1 in Fig. 5A, B, photochemical epoxidation via benzil allowed to reveal 56 *sn*-isomers present in at least two of the three biological replicates and 34 *sn*-compositions that were present in all three replicates in at least one of the two cell lines. The relative *sn*-isomer intensity (intensity of one *sn*-isomer divided by the sum of all signals of the *sn*-isomer pair) is shown in Fig. 5C. In agreement with the recent study by Michael et al. [13], PCs with a small difference between the lengths or DB number of the FA moieties in *sn*−1 and *sn*−2 exhibit subtle differences in *sn*-isomer intensities. In both cell lines, for example, fragments for the *sn*-pairs PC 16:0_18:2 and PC 16:0_16:1 have close to the same intensity ratios. This contrasts with the pairs for PC 16:0_20:4 and PC 18:0_20:4, for which the *sn*−2 location of the PUFA is in large excess to the other isomer, in agreement with the reported selectivity of LPCATs to preferably attach PUFAs to *sn*−2 [4]. But also, differences between the cell lines and occupation preferences of *sn*-sites exist. HeLa cells, for example, have higher PUFA intensities in the *sn*−1 position compared to H9c2 cells. One example is the *sn*-pair PC 18:1_20:3, for which HeLa cells have about the same relative intensity for the two isomers, but the isomer PC 18:1/20:3 is preferred for H9c2 cells (Fig. 5C).

## Conclusion

Photochemical epoxidation of unsaturated lipids by benzil and dissolved oxygen in a flow reactor revealed new possibilities for lipid structure analysis. By epoxidizing unsaturated FAs, glycerolipids (GLs), and GPLs, DB positions are pinpointed in positive- or negative-ion mode across these experimental platforms. This increases the number of lipid classes for which DB positions are accessible in RPLC-MS^n^ of epoxides. An additional benefit of RPLC of epoxidized lipid products compared to non-reacted counterparts is the improved separation capabilities for DB position and *sn*-isomers (Fig. 3). The herein newly described photochemical reaction workflow for lipidomics leads to the isomerization of DB bonds in non-reacted and reacted unsaturated lipids. As DB *E*/*Z* isomers are readily resolved in RPLC of lipids, the benzil-mediated photochemical epoxidation allows annotation of DB geometries without authentic standards for monounsaturated lipids or as long as a saturated FA is paired with a mono- or polyunsaturated FA. Furthermore, the addition of sodium salts to epoxidized unsaturated lipids yielded *sn*-specific fragments in MS^n^ experiments. This opens the possibility to study *sn*-isomerism as showcased for two lipid cell extracts. Therefore, we describe a single derivatization strategy that facilitates assignments of all major aspects of GL and GPL structures, i.e., head group, FA composition, DB position, DB geometry, and *sn*-isomerism, in eukaryotes if appropriate sample preparation or separation methods are employed.

Despite the described benefits, there are areas for improvement. These include the development of custom-made software solutions to identify corresponding fragment ions for *sn*-isomers, increasing sodiated lipid ion intensities for RPLC-MS^n^ in a post-column approach, and the inherent difficulty in assigning *E*/*Z* isomers to specific DB positions if *E* and *Z* DB bonds are in one FA chain or multiple FA moieties with DBs exist. These shortcomings will be addressed in future studies aiming to overcome these challenges. With the presented methodology and potential future improvements, we envision studying the intricate effects of heart diseases on structural features of lipids to link these structural changes to altered metabolic processes after myocardial infarction or heart transplantation.

## Supplementary Information

Below is the link to the electronic supplementary material.

Supplementary file1 Supplementary Material contains additional experimental protocols, MS^n^, RPLC, and validation data. (DOCX 2975 KB)

## Data Availability

The data presented in the manuscript and Supplementary Material is available upon request.
