# Supplementary material for: Comprehensive lipid structure annotation via photochemical epoxidation and mass spectrometry
Source: Anal Bioanal Chem. 2025 Jun 20;417(20):4513–24. doi: 10.1007/s00216-025-05953-6 (PMC12325535; doi:10.1007/s00216-025-05953-6)
Supplement: Supplementary file 1 — Supplementary file1 Supplementary Material contains additional experimental protocols, MSn, RPLC, and validation data. (DOCX 2975 KB) [file 216_2025_5953_MOESM1_ESM.docx]

**Comprehensive Lipid Structure Annotation via Photochemical Epoxidation and Mass Spectrometry**

Jing Yu^1^, Belal Alshaar^1^ and Sven Heiles^1,2*^

^1^Leibniz-Institut für Analytische Wissenschaften - ISAS - e.V., 44139 Dortmund, Germany

^2^Lipidomics, Faculty of Chemistry, University of Duisburg-Essen, 45141 Essen, Germany

* - Corresponding Author, email address: [sven.heiles@isas.de](mailto:sven.heiles@isas.de)

**Supplementary Material**

**Table of content**

| **Supplementary Note 1 - Nomenclature** | **Page S3** |
| --- | --- |
| **Supplementary Note 2 - Methods** | **Page S3-4** |
| **Figure S1** | **Page S5** |
| **Figure S2** | **Page S5** |
| **Table S4 – Reaction optimization** | **Page S6** |
| **Figure S3 – Reaction optimization** | **Page S6** |
| **Figure S4 – Reaction optimization** | **Page S7** |
| **Figure S5 – Reaction optimization** | **Page S7** |
| **Figure S6 – Reaction optimization** | **Page S8** |
| **Figure S7 – Reaction optimization** | **Page S9** |
| **Supplementary Note 3 - Tandem Mass Spectrometry of Photo-Epoxidized Glycerophospholipids** | **Page S9-10** |
| **Figure S8** | **Page S11** |
| **Figure S9** | **Page S12** |
| **Figure S10** | **Page S12** |
| **Figure S11** | **Page S13** |
| **Figure S12** | **Page S13** |
| **Figure S13** | **Page S14** |
| **Figure S14** | **Page S15** |
| **Figure S15** | **Page S16** |
| **Figure S16** | **Page S17** |
| **Figure S17** | **Page S18** |
| **Figure S18** | **Page S19-20** |
| **Figure S19** | **Page S21** |
| **Figure S20** | **Page S22** |
| **Scheme S1** | **Page 23** |
| **Figure S21** | **Page S24-26** |
| **Table S5** | **Page S27-28** |
| **Figure S22** | **Page S29** |

**Supplementary Note 1 – Nomenclature**

**Lipid nomenclature.** To describe lipid structures, the shorthand nomenclature of LIPIDMAPS is used in this manuscript. For example, PC 16:0_18:1(9Z) indicates a phosphatidylcholine (PC) lipid containing C16 and C18 fatty acyl chains. The number after the colon refers to the degree of unsaturation of each fatty acyl chain, the location of the C=C bond, 9, is counted from the carboxyl side of fatty acyl chain. The *Z* label describes the geometry of the C=C bond. If the C=C geometry is not available from experiments, a Δ sign is added before the location C=C bond location. The underscore (“_”) indicates that the *sn*-position of these two fatty acyl chains is not identified. If *sn*-positions are identified, a “/” is used between the FA moieties. The *sn*-1 FA chain is placed before the “/”, the *sn*-2 chain behind the “/”.For epoxidized lipids, for example PC 16:0_18:0;9Ep(cis), the sign “Ep” accounts for epoxide ring, the number 9 refers the ring location, the *trans* and *cis* label will be used to indicate stereoisomers.

**Supplementary Note 2 – Methods**

**Mass spectrometry and Reversed Phase Liquid Chromatography**

Lipids were separated by reverse phase liquid chromatography (RPLC) on Vanquish Horizon System (Thermo Fisher Scientific, Dreieich, Germany) equipped with a YMC C18 column (0.075 mm x 150 mm, 1.9 µm, Dinslaken, Germany). Lipids were separated by gradient elution with solvent A (acetonitrile/water, 1.1, v/v) and B (isopropanol/acetonitrile/water, 85:10:5, v/v) both containing 5 mM ammonium formate and 0.1% (v/v) formic acid. Separation was performed at 50°C with flow rate of 0.3 mL/min using different gradients for FAs, GPL standards, and lipid extracts. More details on the gradients are listed in **Table S1-S3**.

RPLC was coupled on-line to one of two orbitrap mass spectrometers (Velos Pro and Eclipse, Thermo Fisher Scientific, Bremen, Germany) equipped with a heated electrospray ionization (HESI) source.

In positive-ion mode, the ion source voltage was 3.5 kV, the source temperature was 370 °C, the sheath gas flow rate was 40, the aux gas flow rate was 10, the sweep gas flow rate was 1, the capillary temperature was maintained at 300 °C, and the AGC target was set to 1 x 10^6^ ions for full MS scans and 5 x 10^4^ ions for SIM and MS^n^ scans.

In negative-ion mode, the ion source voltage was -3.2 kV, while all other parameters remained identical to those used in positive-ion mode except for the AGC target for full MS scans, which was set to 3 x 10^6^ ions.

Data acquisition for lipid identification was performed in this manuscript by data dependent acquisition mode (DDA). DDA parameters are: Survey scan resolution 120k (at m/z 200), RF 35%, maximum injection time 100 ms, scan range of *m*/*z* 200-1200.

Data dependent MS/MS scan were acquired with a resolution of 30k, the maximum injection time was set to 150 ms, isolation window was set to ±0.5 *m*/*z*, activation type CID and NCE of 35. All isotopes and charge states >1 were excluded. All data were acquired in profile mode.

**Table S1**: Gradient for FAs.

| Time (min) | %B |
| --- | --- |
| 0 | 15 |
| 18 | 86 |
| 25 | 95 |
| 29.9 | 95 |
| 30 | 15 |
| 34 | 15 |

**Table S2:** Gradient for GPL standards.

| Time (min) | %B |
| --- | --- |
| 0 | 10 |
| 3 | 25 |
| 10 | 50 |
| 19 | 75 |
| 28 | 95 |
| 30 | 95 |
| 30.1 | 10 |
| 34 | 10 |

**Table S3**: Gradient for all extracts.

| Time (min) | %B |
| --- | --- |
| 0 | 10 |
| 3 | 25 |
| 10 | 50 |
| 19 | 75 |
| 28 | 95 |
| 38 | 95 |
| 38.1 | 10 |
| 41 | 10 |

**Data Processing**. All annotations presented in this work rely on accurate mass measurements obtained from MS^1^ and MS^n^ spectra. Potential annotations were accepted within a mass error tolerance of ±5 ppm for MS^1^ and ±10 ppm for MS^n^. For epoxidized lipids and corresponding fragments that are not included in the LipidMaps database, sum formulae with low ppm were manually assigned and used to infer potential fragmentation pathways.

Following the identification of reaction and fragmentation products, customized databases were constructed. Subsequent ion searches were conducted using the same mass error tolerances as described above. Whereas mass spectrometric data exhibits mass errors below ±5 ppm for MS^1^ and ±10 ppm for MS^n^, only two decimal places are reported in the manuscript for simplicity.

QualBrowser was utilized for generating mass spectra and extracting ion chromatograms. Lipid annotations prior to epoxidation was performed using Liporstar2 (Mass Analytica, Spain). All figures and mass spectra were generated using Python or Origin and subsequently refined in Microsoft PowerPoint or Adobe Illustrator. The epoxidation reaction yield was calculated according to $\%yield=\frac{I_{\mathrm{epoxidized}}}{I_{\mathrm{epoxidized}}+I_{un-epoxidized}}*100\%$. $I$ is the peak intensity from shotgun MS measurements. For LCMS measurements, the reaction yield was calculated by the same formula but with MS intensity replaced by LC peak areas.

**Sn-Isomers and Isobaric Overlap**

IsoPure standards were used in experiments with PEs and PCs that have less than 3 % isomeric impurities. Whereas lipids with the composition 16:0/18:0;Ep always showed a signal at *m*/*z*361.27 with about 5-10% intensity relative to *m*/*z* 291.23, tandem MS spectra of 18:0;Ep/16:0 exclusively yielded *m*/*z* 361.27 (**Figure S17**). The apparent impurity for 16:0/18:0;Ep stems from the isobaric overlap from the neutral loss of FA 16:0 which is not the case for 18:0;Ep/16:0 for which the neutral FA loss does not overlap with the 5-membered ring cleavage. Isobaric overlap can only occur if the FA moieties differ by two CH_2_ groups and one DB. In this case, relative ion abundances are systematically influenced by one of the two isomers by 5-10% relative intensity.


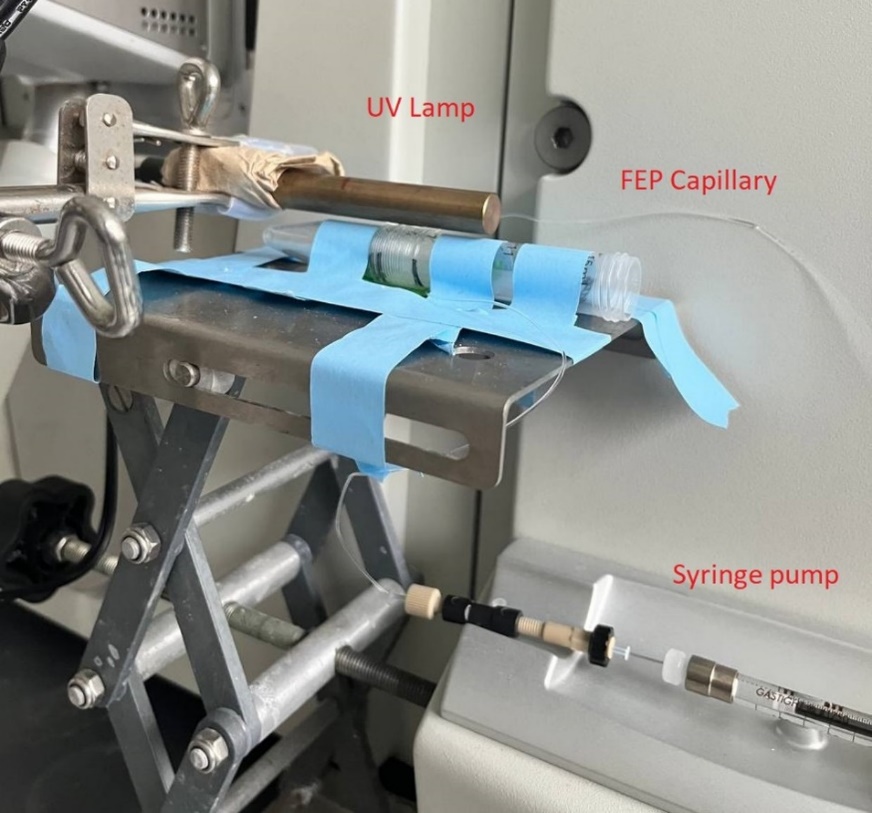


**Figure S1.** Photograph of the flow reactor. The photo derivatization flow reactor was built by coiling a 1.5 m long fluorinated ethylene propylene (FEP) capillary (1/32" OD x 0.38mm ID) around a 15 mL volume falcon tube. A low-pressure mercury pencil lamp (UVP, Upland, CA, USA) with brass shield and a band pass filter was placed about 5 mm away from the outer surface of capillary coil providing UV light at an emission maximum of 254 nm.


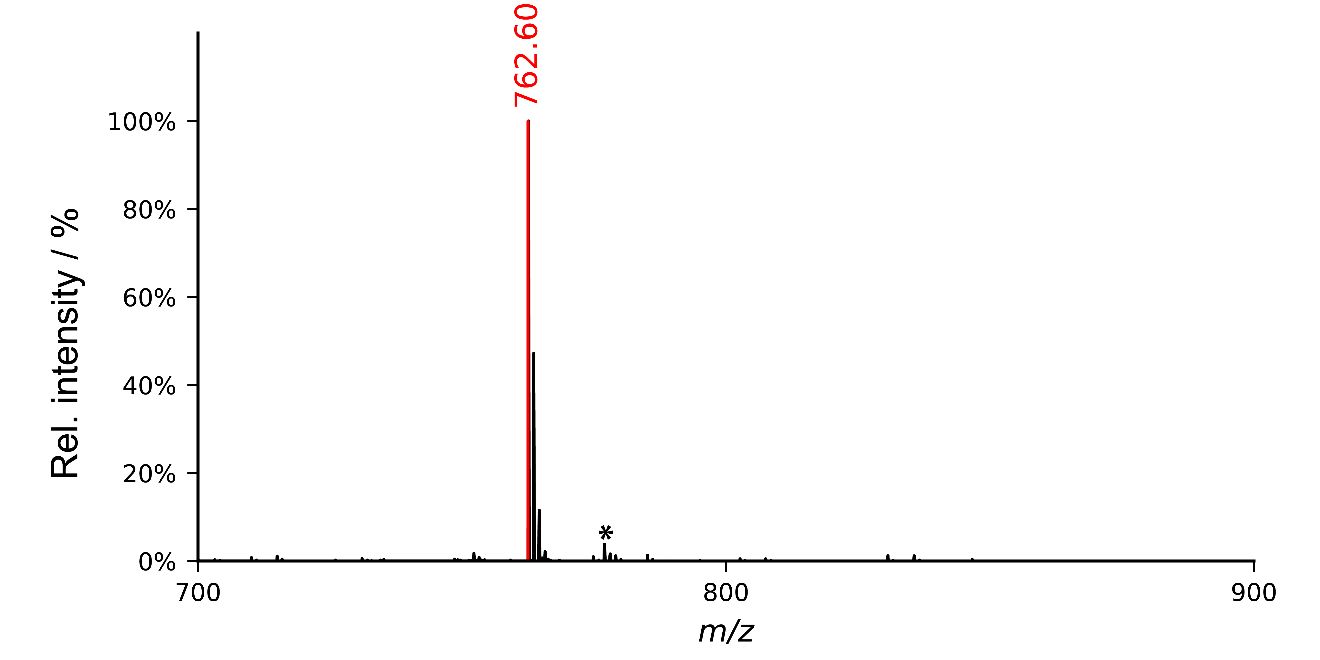


**Figure S2**: The MS^1^ spectrum of PC 16:0/18:0 exposed to benzil showed no evidence of epoxidized adducts or oxidation, in contrast to the spectra of unsaturated species. The signal marked with an * at m/z 776.58 was identified as a lipid impurity in the standard. Additionally, the MS/MS spectra of this impurity did not exhibit water loss peaks or other indication.

**Table S4**: Reaction Optimization - Effect of solvent composition and additives on the epoxidation yield and side reactions.

| **Solvents** | **1mM benzil + additives** | **Intensity of epoxide signals relative to precursor** |
| --- | --- | --- |
| Acetonitrile |  | Below 1% |
| Acetonitrile | 1M ammonia | 50%, no side reaction |
| Acetonitrile | 1M hydrogen peroxide | 30%, pronounced side reactions |
| Acetonitrile | 10mM Boc-hydroxylamine acid | None |
| Methanol |  | Below 1% |
| Tetrahydrofuran |  | None |
| Isopropyl alcohol |  | None |
| Dichloromethane |  | None |
| Acetonitrile/water |  | > 100% |


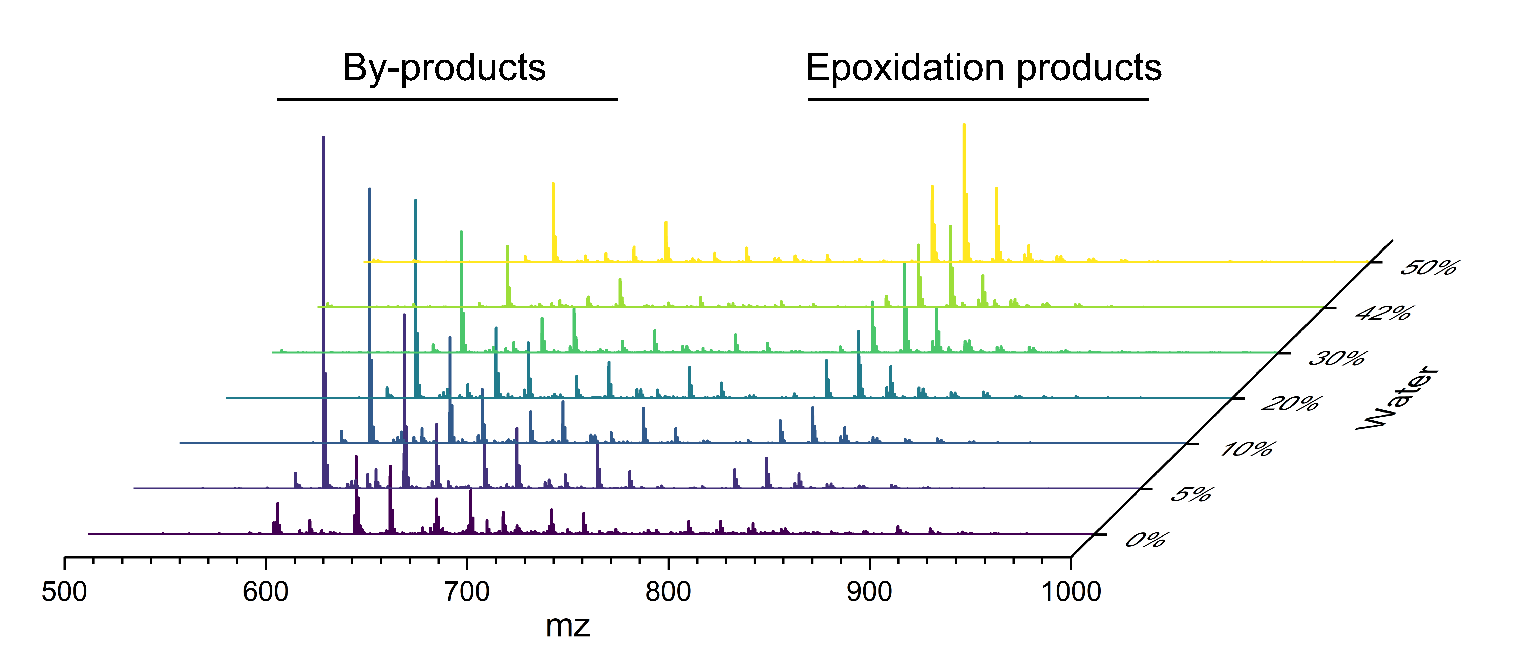


**Figure S3**: Reaction Optimization *-* Investigating the effect of water content relative to acetonitrile the on epoxidation and side reactions yield for PC 16:0/20:4 upon benzil-mediated photoreaction. Side reactions are minimized when increasing the water content above 50 % after which some lipids stopped to dissolve.


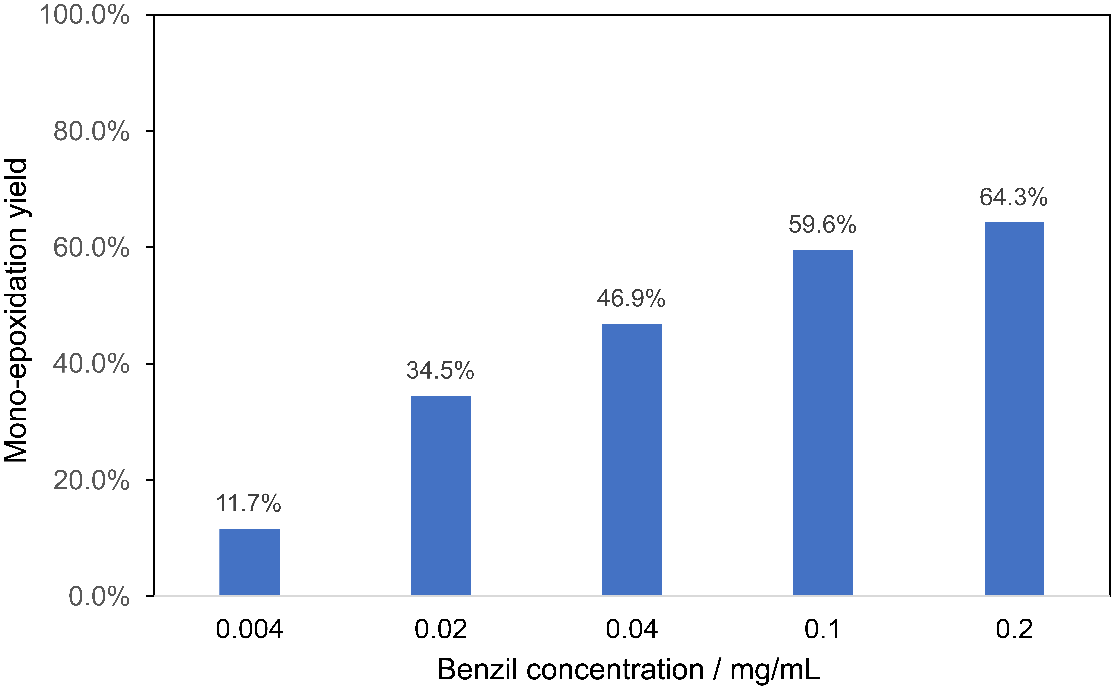


**Figure S4**. Reaction Optimization *-* Impact of the benzil concentration on mono-epoxidation yield: 0.01 mg/mL PC 16:0/20:4 with varying benzil concentration, injection rate set as 40 µL/min, total injection volume 500 µL/min. Above 0.2 mg/mL benzil concentration side reaction products increase. Therefore, 0.2 mg/mL were selected as concentration with the highest yield of mono-epoxidation and at the same time low levels of side reactions.


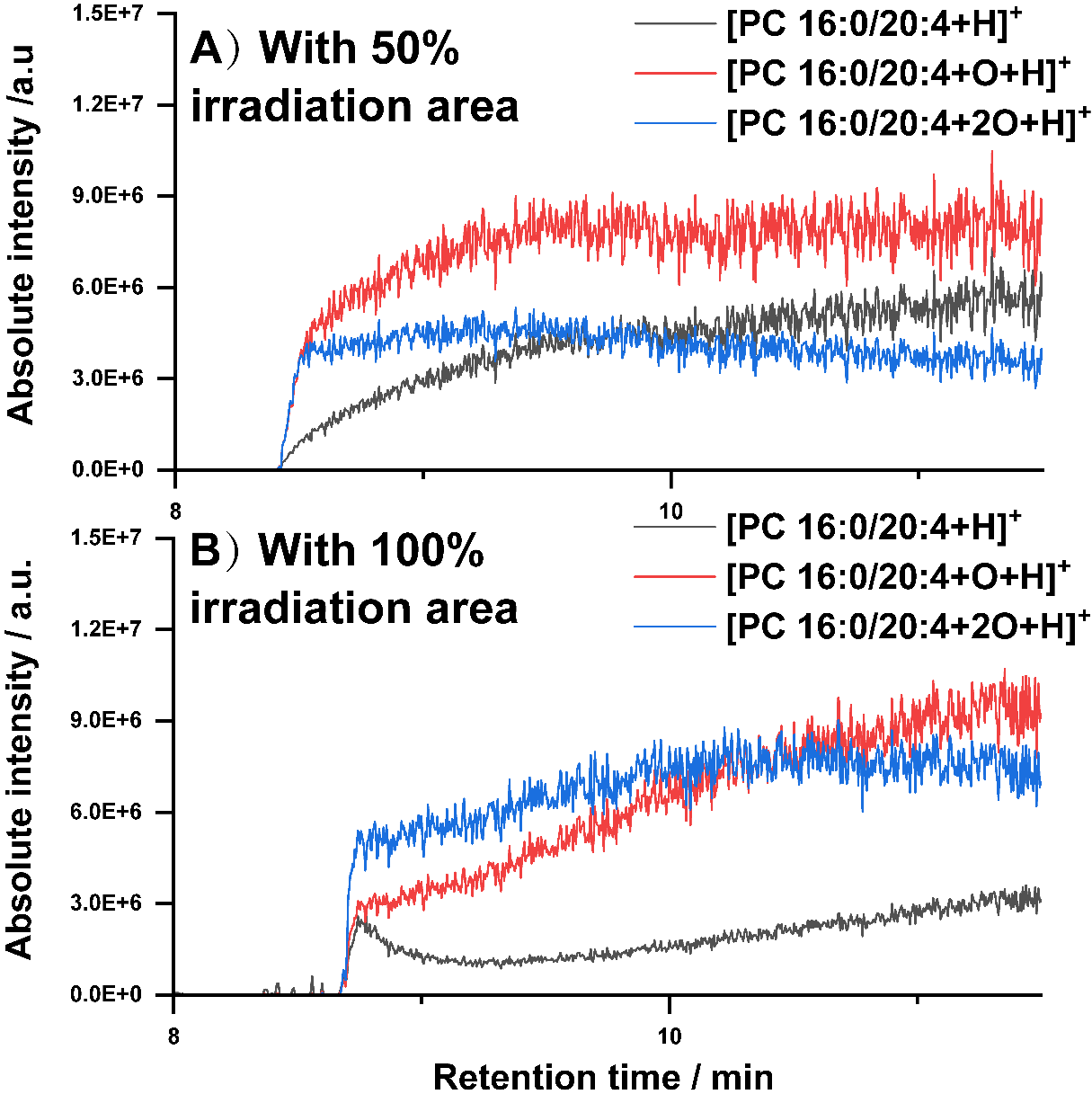


**Figure S5:** Reaction Optimization - *m*/*z* trace of PC 16:0/20:4 and the mono as well as doubly epoxidized products. A) The effective reaction time was reduced by a factor of two compared to B) by covering 50% of the flow reactor with a piece of aluminum foil. B) Irradiation of the entire flow reactor. The ratio between mono- and doubly epoxidized reaction products is influenced in flow reactors by adjusting the irradiated part of the reactor and as a consequence changing the effective reaction time. Because the overall reaction yield is maximized when irradiating the entire reactor with light, condition B) was used throughout the manuscript. In the future, adjusting reaction conditions by altering the effective length which is irradiated is, however, possible.


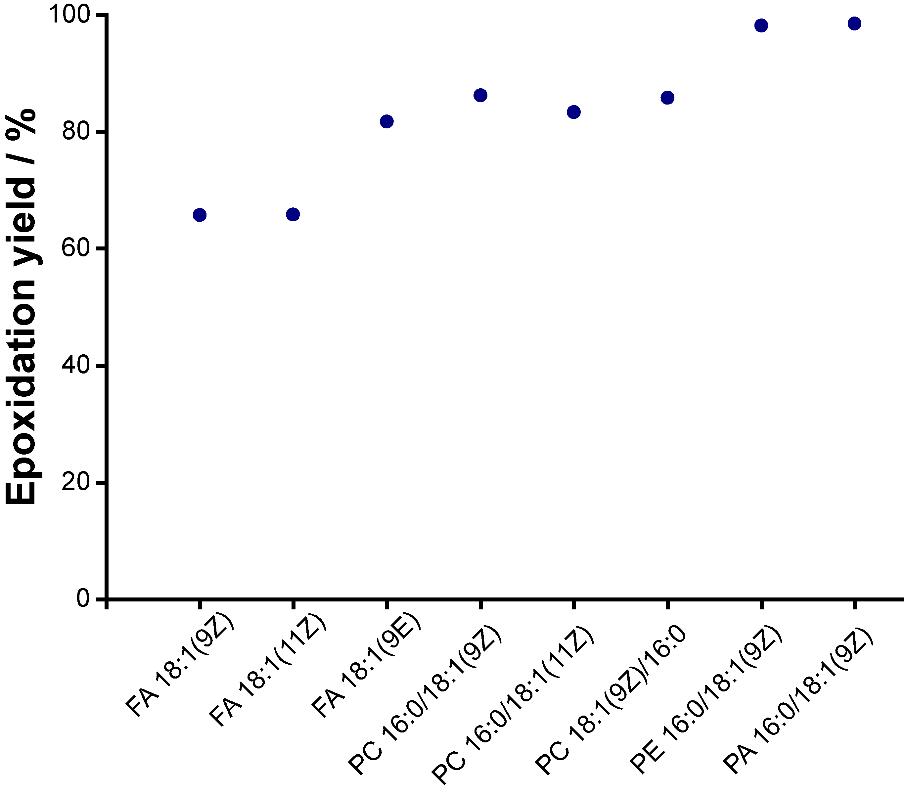


**Figure S6**: Reaction Optimization - Epoxidation yield (based on LC-MS peak areas) for different lipid standards employing the optimized conditions. These are mentioned in the manuscript and are as follows: 500 µL mixture of lipid standard 0.01 mg/mL and 0.2 mg/mL benzil in acetonitrile: water (1:1, v/v) inject into flow reactor under UV irradiation at 40 µL/min.

**
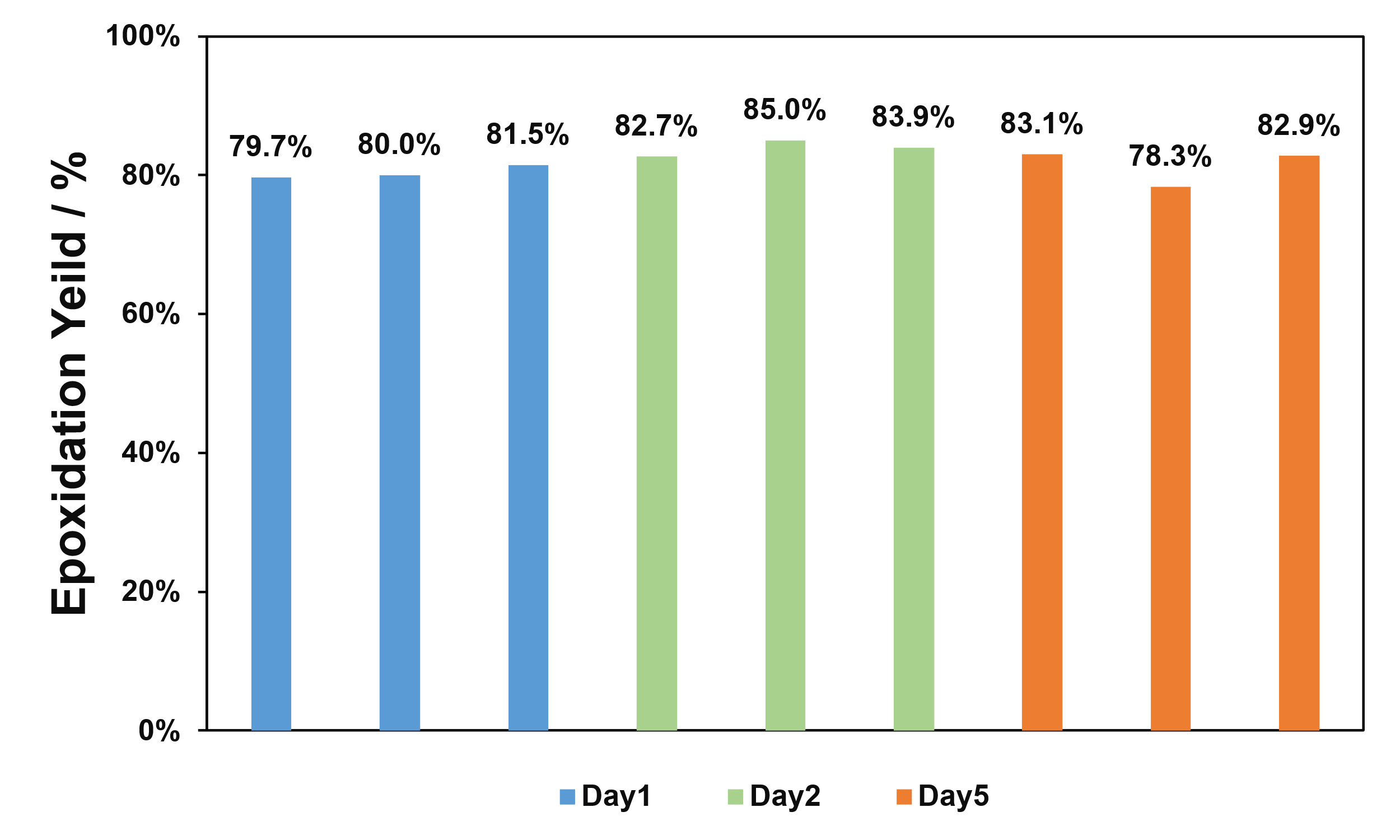
**

**Figure S7**. Reaction Optimization - The inter-day and intra-day reproducibility of the epoxidation yield (measured by intensities) was assessed using optimized conditions: a 500 µL mixture of lipid standard PC 16:0/18:1(9Z) at 0.01 mg/mL and 0.2 mg/mL benzil in a 1:1 (v/v) acetonitrile: water solution. This mixture was injected into a flow reactor and exposed to UV irradiation of the entire reactor at a flow rate of 40 µL/min. The average value and standard deviation are 81.9 % and ±2.1 %, respectively. Maximum changes of room temperature, humidity, and pressure are ± 1.5 °C, ± 20 °C and ± 25 mbar, respectively, in these measurement intervals as monitored by our lab regulation system. Therefore, the reaction outcome is insensitive to these pressure and temperature changes within the reported uncertainties.

**Supplementary Note 3 - Tandem Mass Spectrometry of Photo-Epoxidized Glycerophospholipids**

After establishing the relevant aspects of the mechanism of benzil-mediated photochemical epoxidation for lipid analysis, epoxidized authentic standards were activated via CID to identify structure-selective fragments. The results for FAs are discussed in **Figure S8** and results for GPLs are shown in **Figure S10** for PE 16:0/18:1(9Z) and **Figure S15** for other PEs and other lipid classes. First, we investigated the MS^2^ of protonated compounds. Consistent with previous reports for other lipid classes,^1^ CID of epoxidized PE 16:0/18:0;9Ep results in pronounced head group loss ions at *m*/*z* 593.51 as well as additional fragments at around *m*/*z* 460 (**Figure S10B**). Two of these signals, i.e., *m*/*z* 451.38 and *m*/*z* 467.37, are consistent with epoxide fragmentation enabling DB position assignment. But also fragments stemming from FA loss are observed (**Figure S10B**) revealing the FA composition. Similar results are obtained in an MS^3^ assay for negative ions (**Figure S16**) as reported before.^2^ This indicates, that the DB position of benzil-epoxidized GPLs is obtained from positive-ion mode MS^2^ or negative-ion mode MS^3^ results consistent with recent reports.^1^

Next, we fragmented sodiated GPLs after epoxidation and results are shown in **Figure S10C,D** and **Figure S17**. To the best of our knowledge sodiated epoxidized GPLs other than PCs were not investigated before. A dominant fragment ion signal in the MS^2^ is the neutral loss of the head group at *m*/*z* 615.50 (**Figure S10C**). Consistent with PB results by Ma and coworkers, fragmentation of *m*/*z* 615.50 in a MS^3^ experiment (**Figure S10D**) yields structure-selective fragments.^3^ The signal at *m*/*z* 321.24 is consistent with positively-charged sodiated FA 18:1;O. The signal at *m*/*z* 489.35 is in line with the cleavage of the epoxide of a sodiated precursor enabling DB position assignment. However, the second signal of the epoxide cleavage is absent or only present in very low abundance. Additionally, a signal at *m*/*z* 291.23 (**Figure S10D**) is observed. In complete analogy to PB-MS^3^ fragments reported by Cao *et al*., *m*/*z* 291.23 is attributed to the loss of the sodiated epoxidized FA 18:1 moiety from the five-membered ring intermediate formed upon neutral head group loss (**Figure S10C**). Because this fragment can only be formed after formation of the five-membered ring intermediate (**Figure S10C**) and multiple groups have shown that this dominantly occurs with the involvement of *sn*-2 FAs of the precursor ion,^4, 5^ this fragment is *sn*-isomer selective. This is also consistent with its absence in the MS^3^ of sodiated PE 18:0;9Ep/16:0 (**Figure S17**). Because the unsaturated FA in PE 18:0;9Ep/16:0 is located at *sn*-1, the corresponding MS^3^ contains a fragment at *m*/*z* 361.27 indicating the loss of a sodiated FA 18:0;9Ep moiety from the *sn*-1 site (**Figure S17**). Plausible fragmentation pathways consistent with MS^2^ and MS^3^ results are given in **Figure S18**. Similar results are obtained for MS^4^ experiments of sodiated GPLs with improved signal-to-noise for PCs compared to MS^3^ (**Figure S19**). Taken together, these results demonstrate that an MS^n^ assay of benzil-mediated epoxidation of sodiated GPLs can yield *sn*-position, DB position, head group, and FA composition.


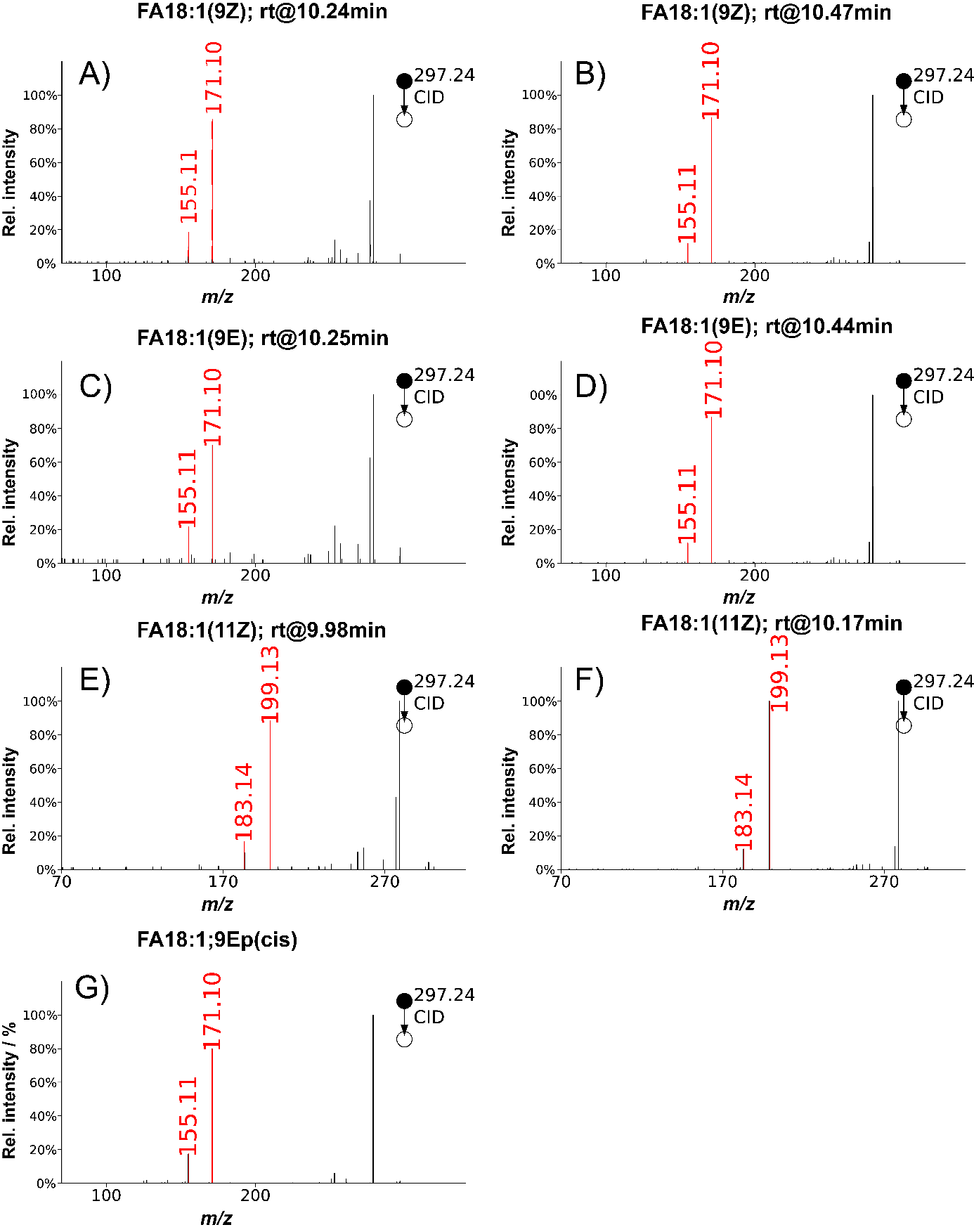


**Figure S8**: LC-MS^2^ of FA standards and epoxidation products in negative-ion mode.


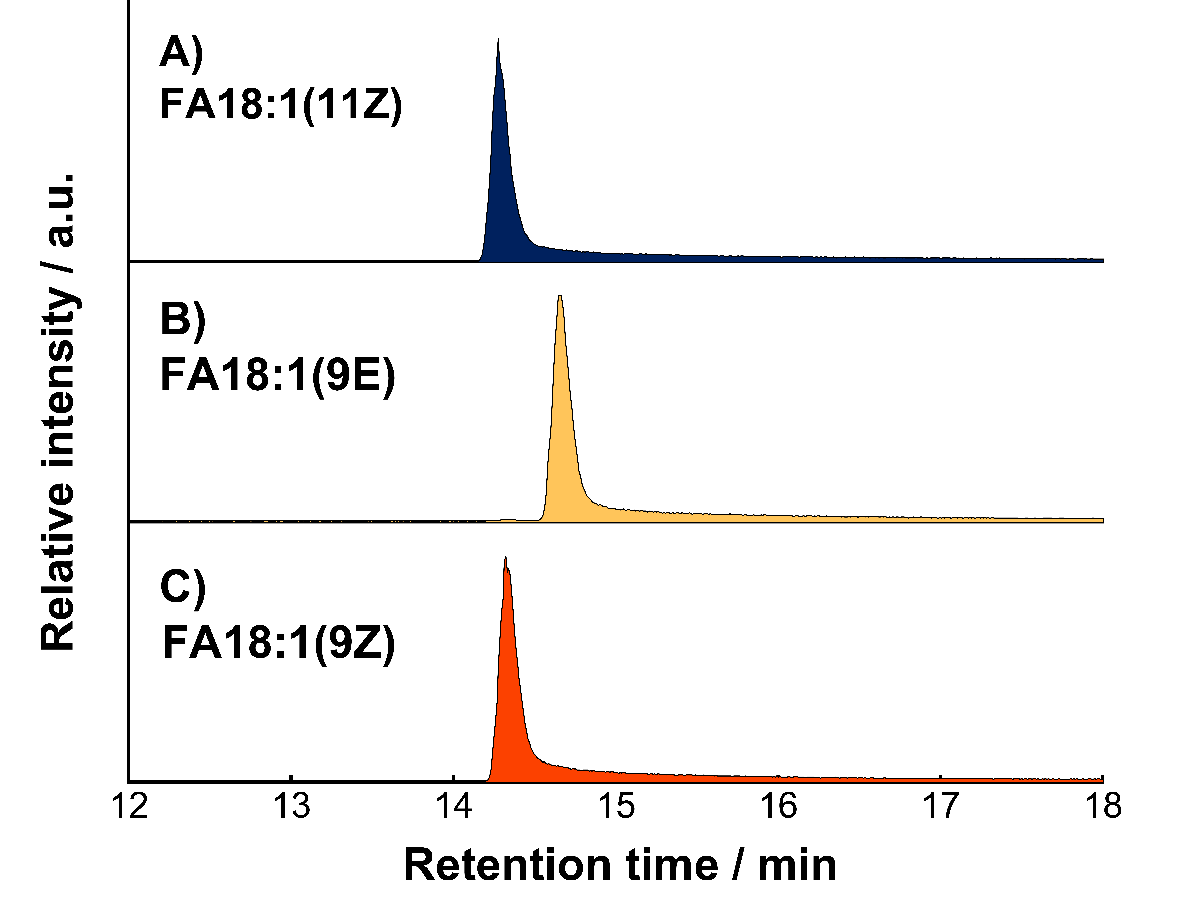


**Figure S9:** HPLC profile of authentic FA 18:1 standards prior to benzil reaction.

**
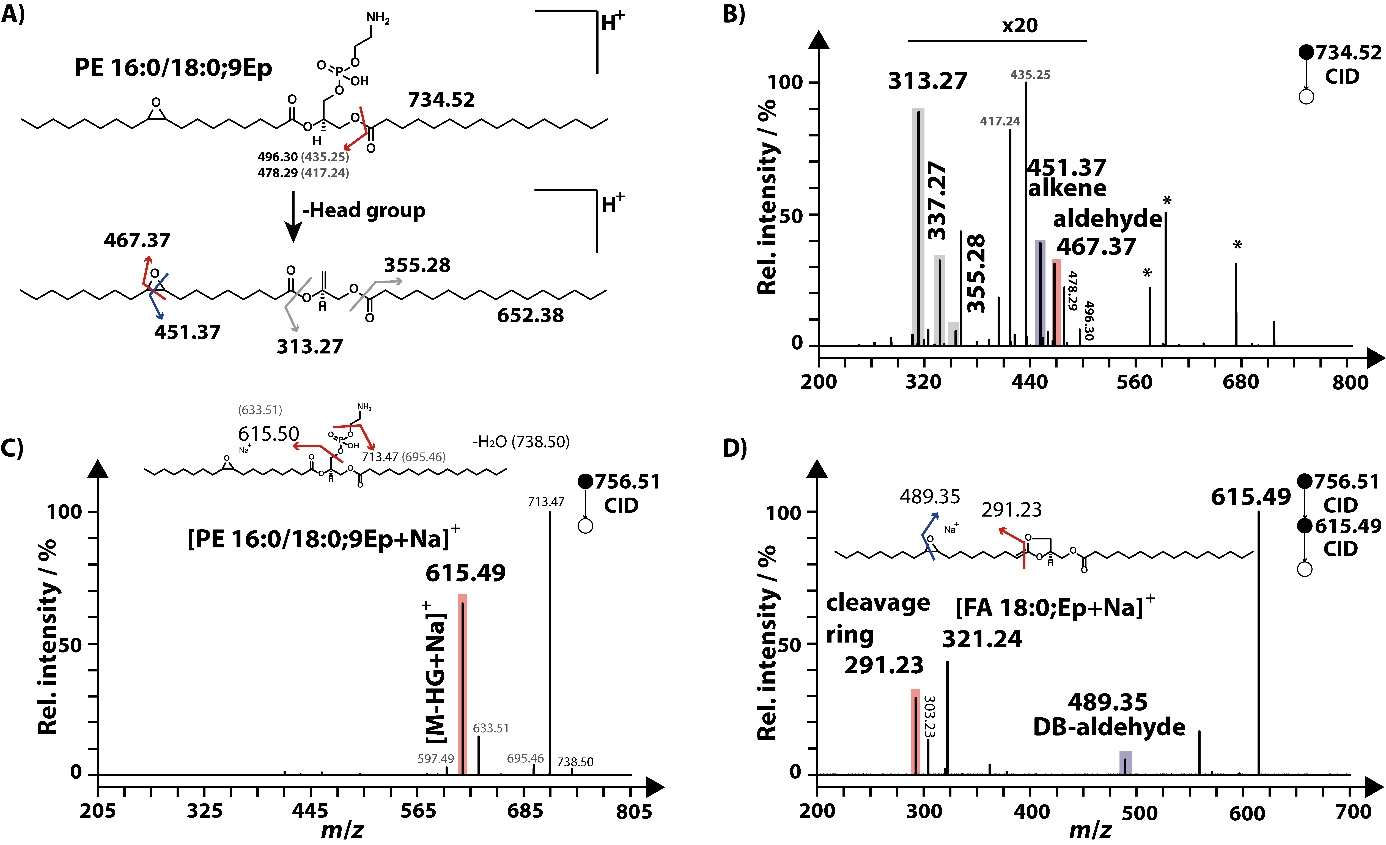
Figure S10**: Tandem mass spectrometric results for PE 16:0/18:1(9Z) after benzil-mediated epoxidation. A) Possible fragment ions of protonated PE 16:0/18:0;9Ep, 496.30,478.29 are produced from fatty acyl chain loss, 435.25, 417.24 are from their further cleavage of P-O bond in the head group. B) MS^2^ of protonated PE 16:0/18:0;9Ep in positive-ion mode; C) MS^2^ of [PE 16:0/18:0;9Ep+Na]^+^ and D) MS^3^ after neutral head group loss. Some ions and their m/z values are highlighted and possible fragment ion structures as well as cleavage sites are indicated. Peaks labeled with a * indicate fragments associated with head group fragmentation.


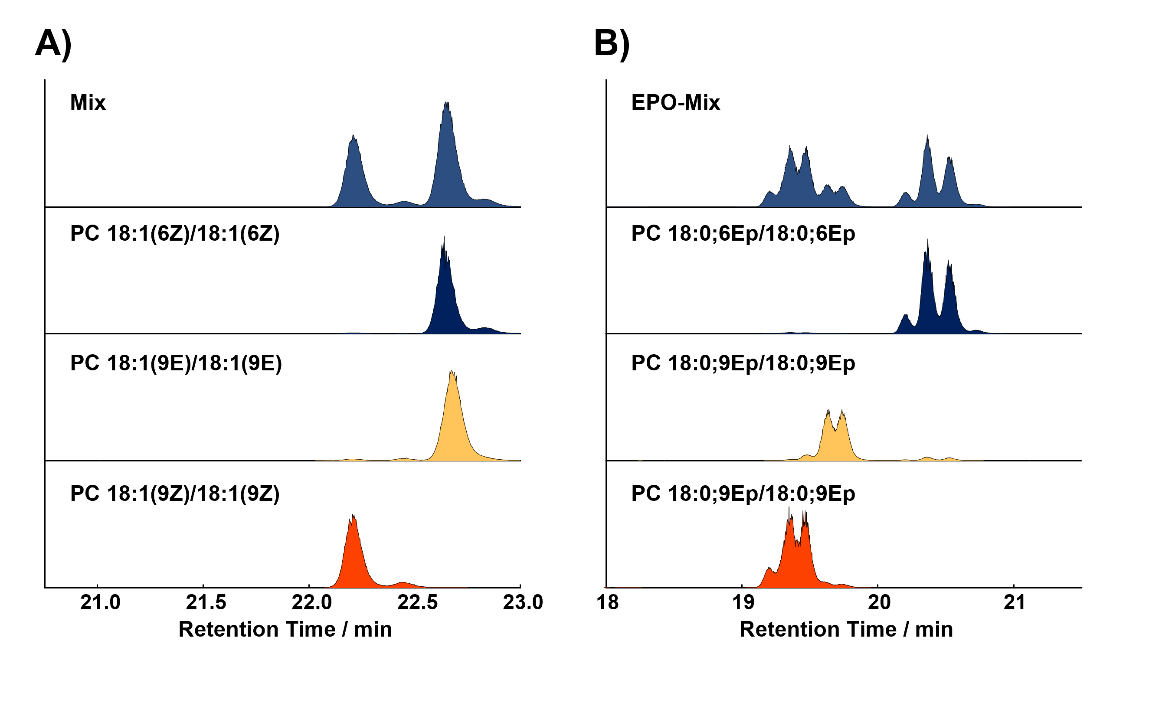


**Figure S11.** RPLC profile of PC 36:2 after epoxidation (EIC). A) PC 36:2 profile after epoxidation. B) Profile of PC 36:2, epoxidation products profile after reaction.


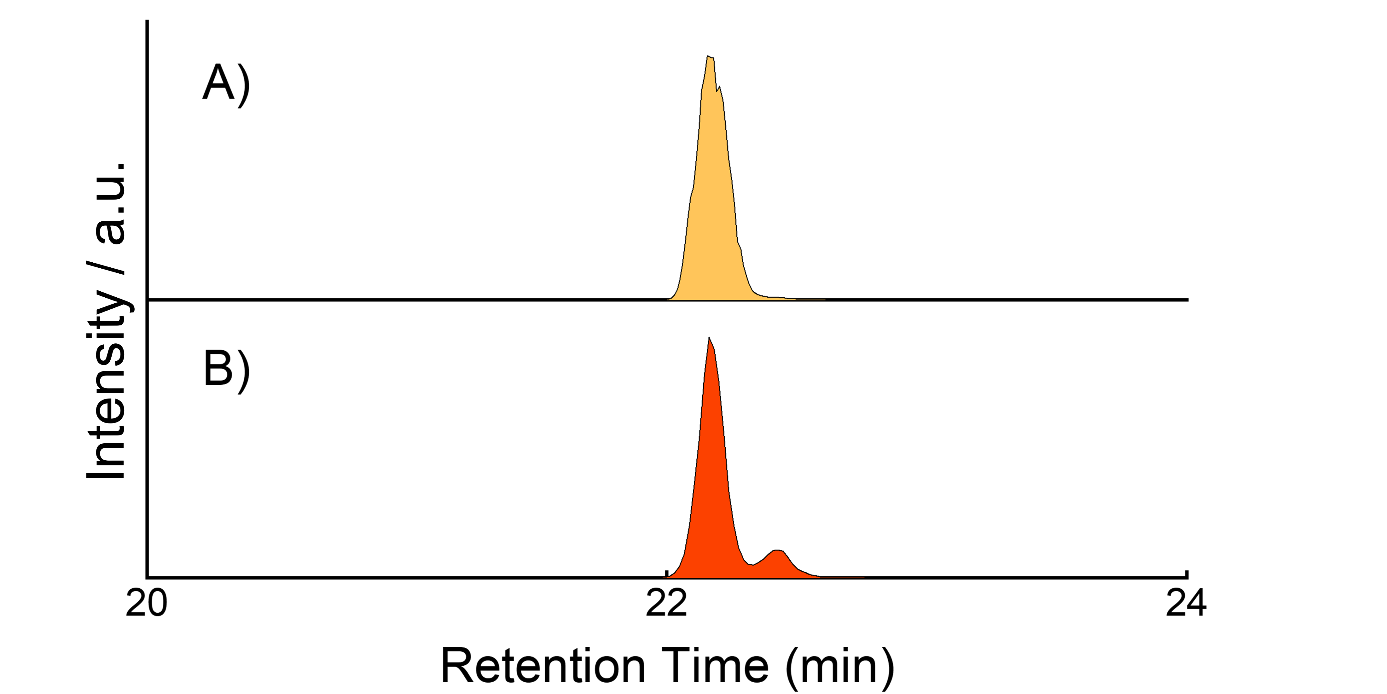


**Figure S12.** RPLC EIC profile of PC 34:1 without epoxidation. A) PC 34:1 mixture prior to epoxidation. B) PC 34:1 mixture after epoxidation.


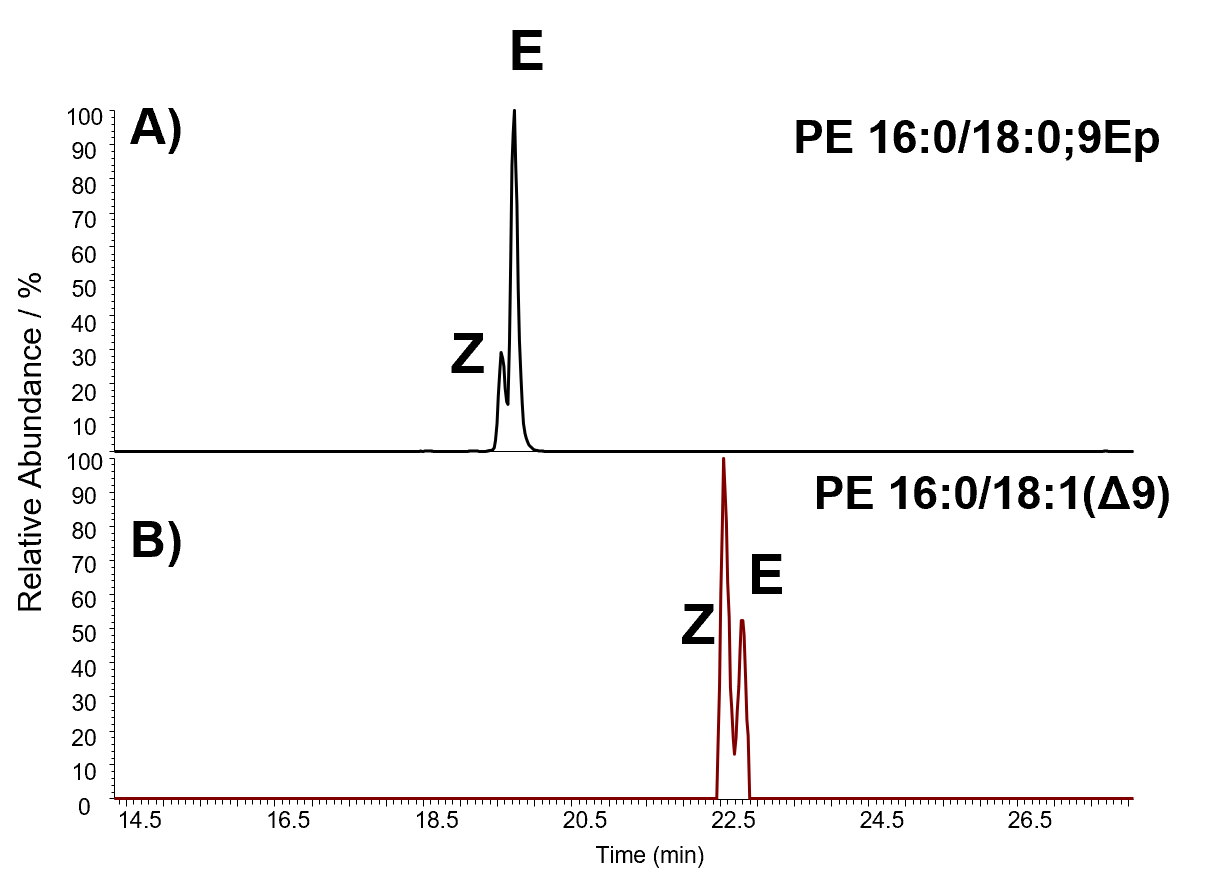


**Figure S13.** [M+H]^+^ trace of the epoxidized product and the post-reaction reactant profile of PE 16:0/18:1(9Z). (A) Extracted ion chromatogram (EIC) of the mono-epoxidized product. (B) EIC profile of the reactant after the reaction.


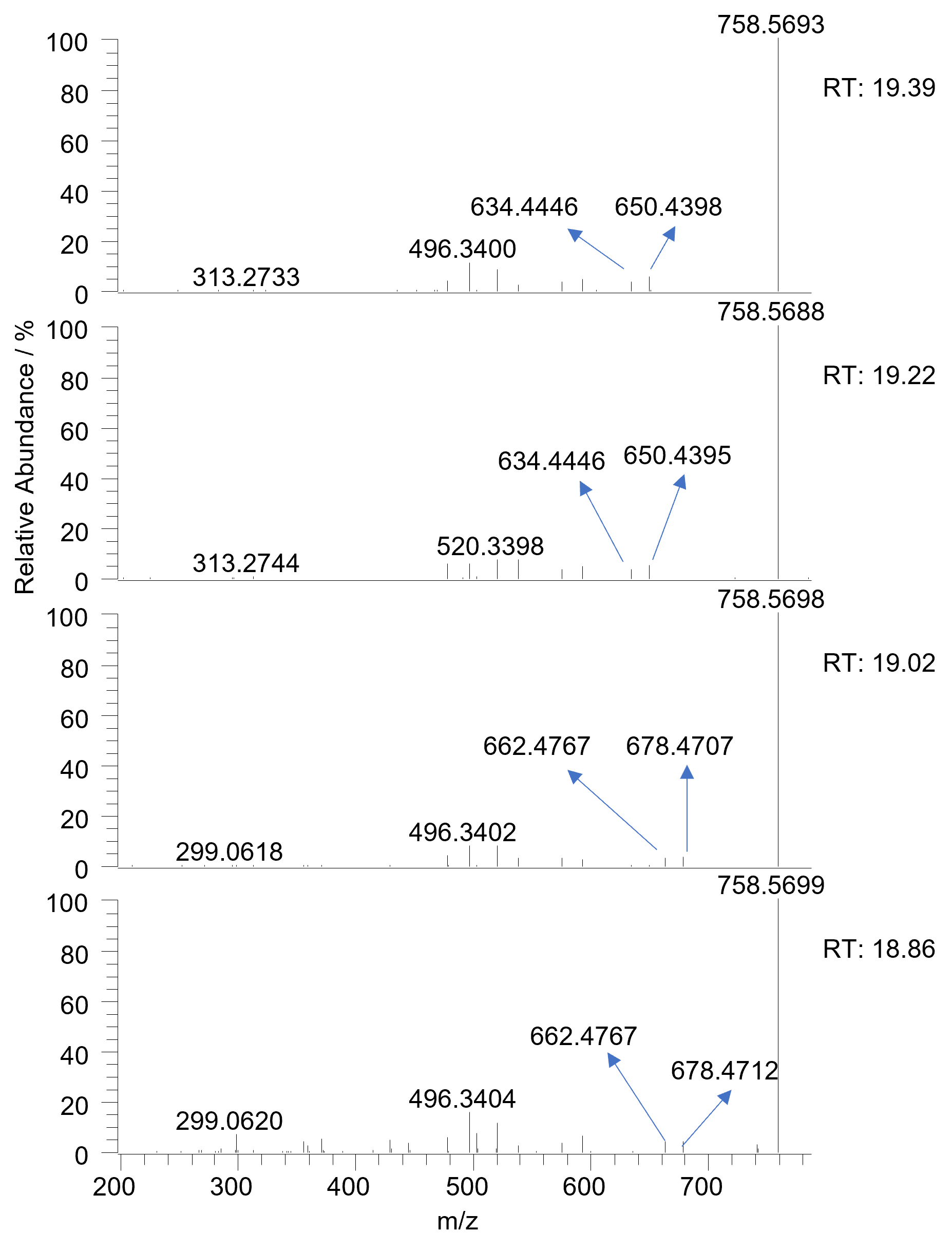


**Figure S14.** [M+H]^+^ MS^2^ of epoxidized PC 34:1 at different retention times.


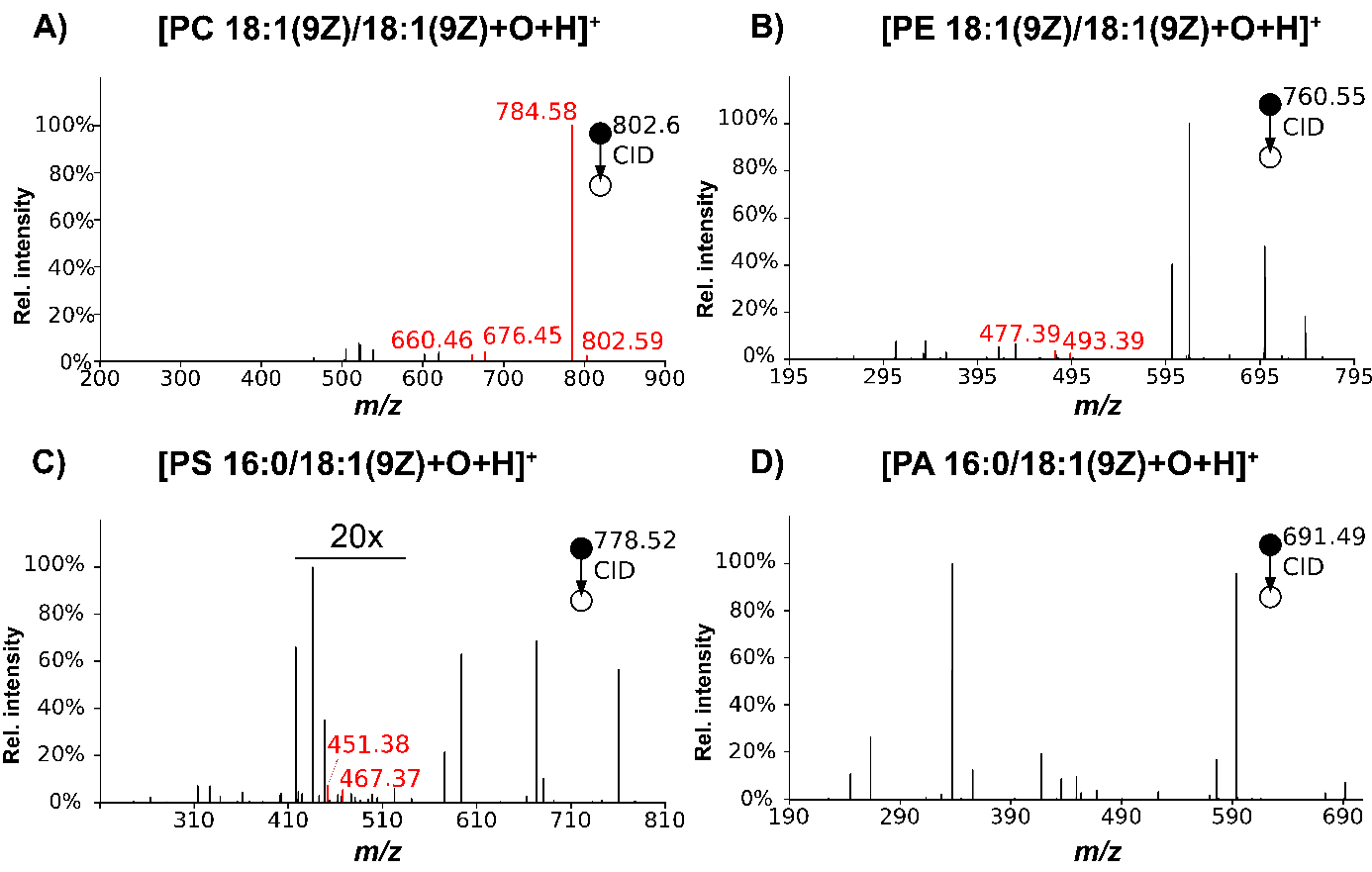


**Figure S15.** LC-MS^2^ spectra of protonated after glycerophospholipids benzil-induced epoxidation. LC-MS^2^ spectrum A) of PC 16:0/18:1(9Z); B) PE 16:0/18:1(9Z); C) PS 16:0/18:1(9Z); D) PA 16:0/18:1(9Z). Some peaks are highlighted including C=C position specific peaks.


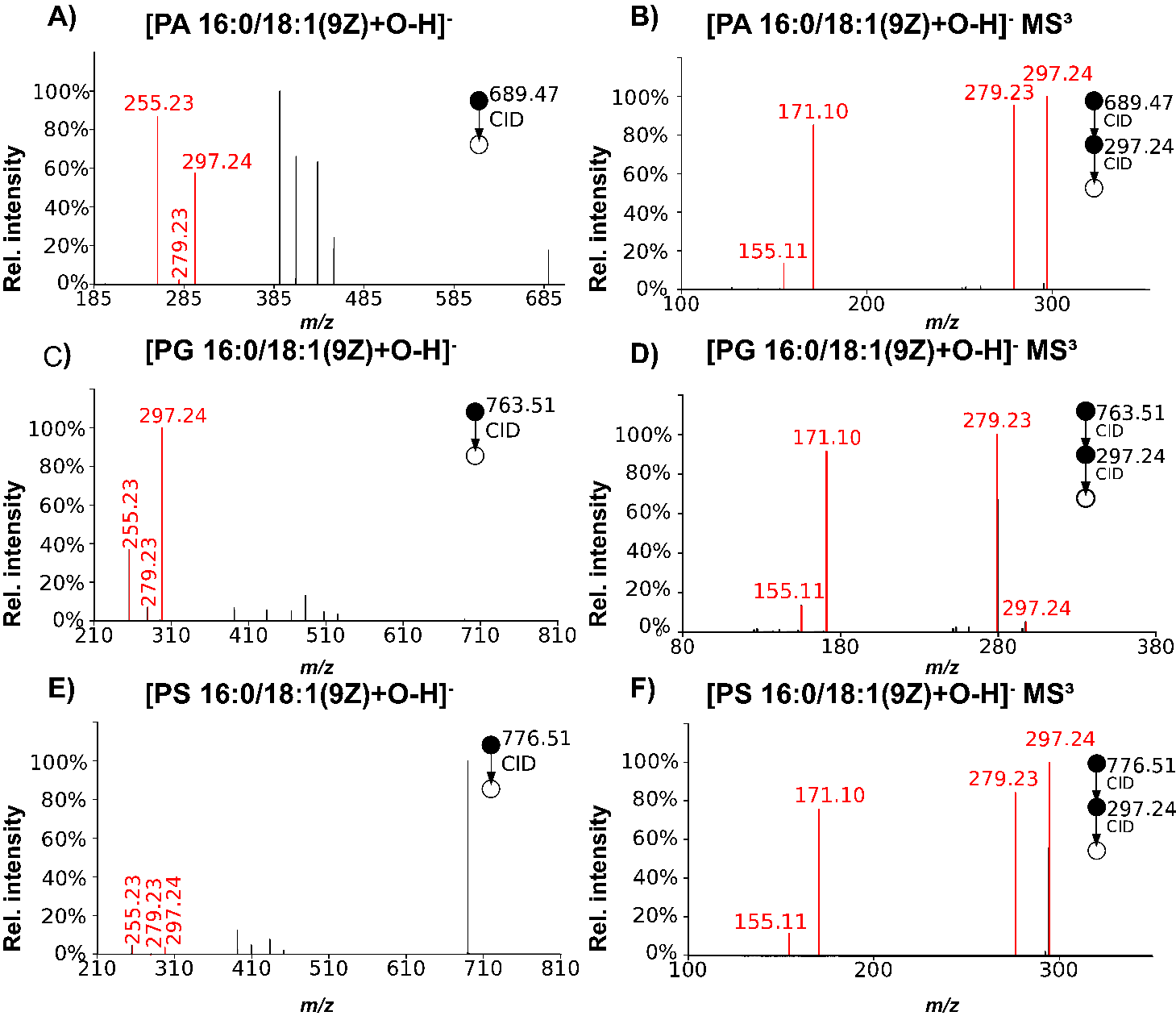


**Figure S16.** Epoxidized unsaturated glycerophospholipids MS^n^ spectra in negative-ion mode.


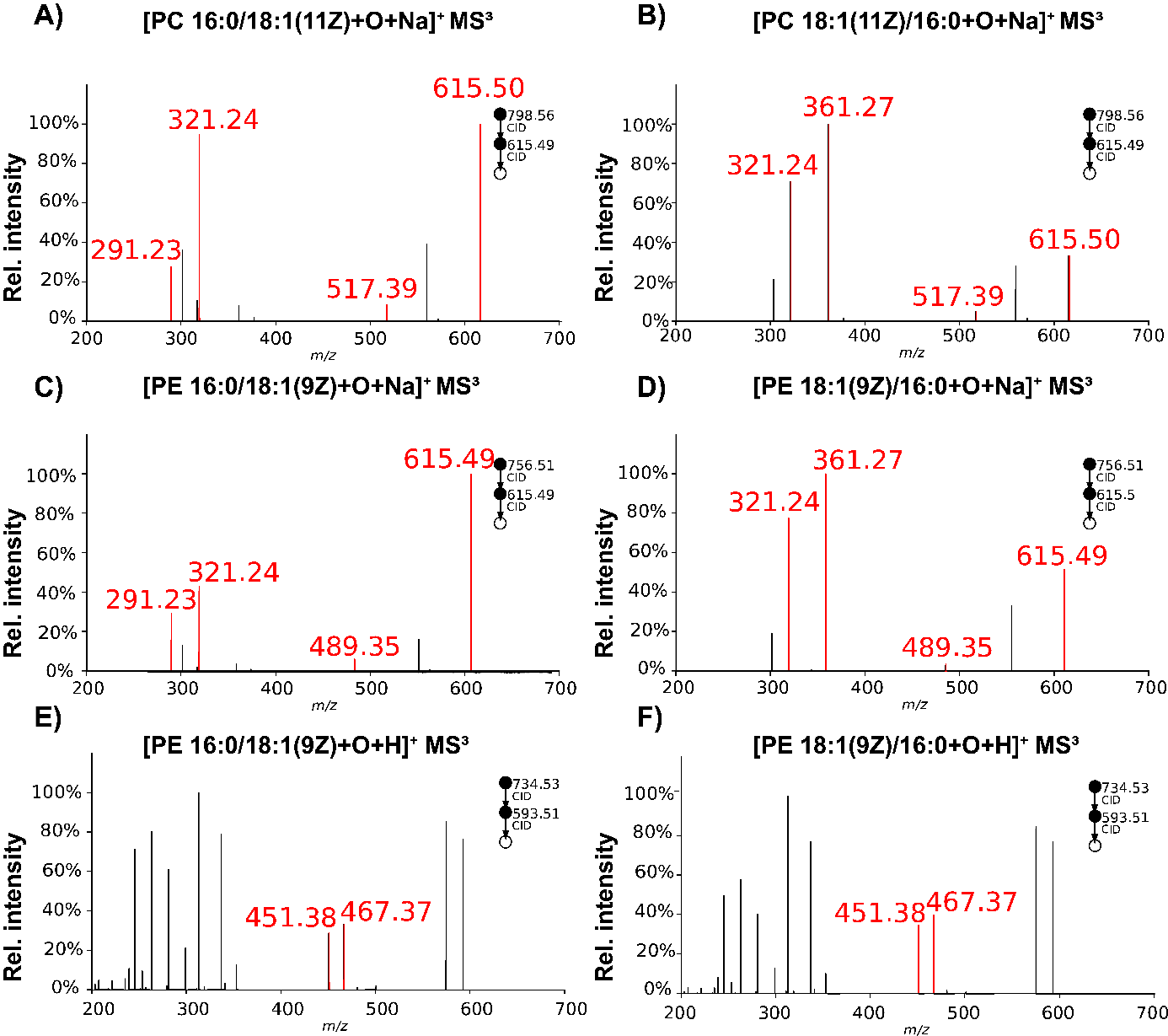


**Figure S17.** Epoxidized unsaturated glycerophospholipids MS^n^ spectra in positive-ion mode of sodiated (A-D) and protonated (E-F) compounds.


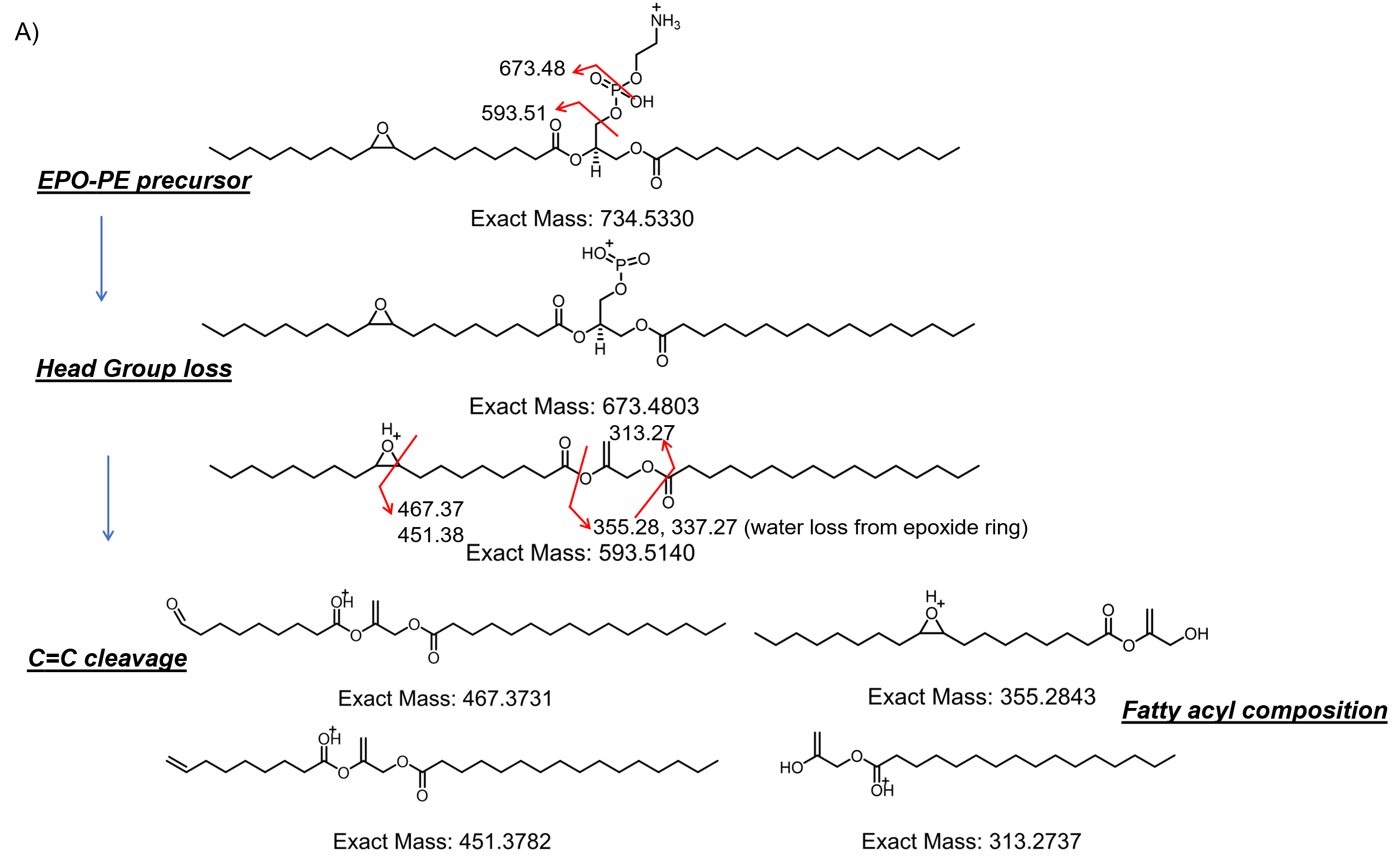

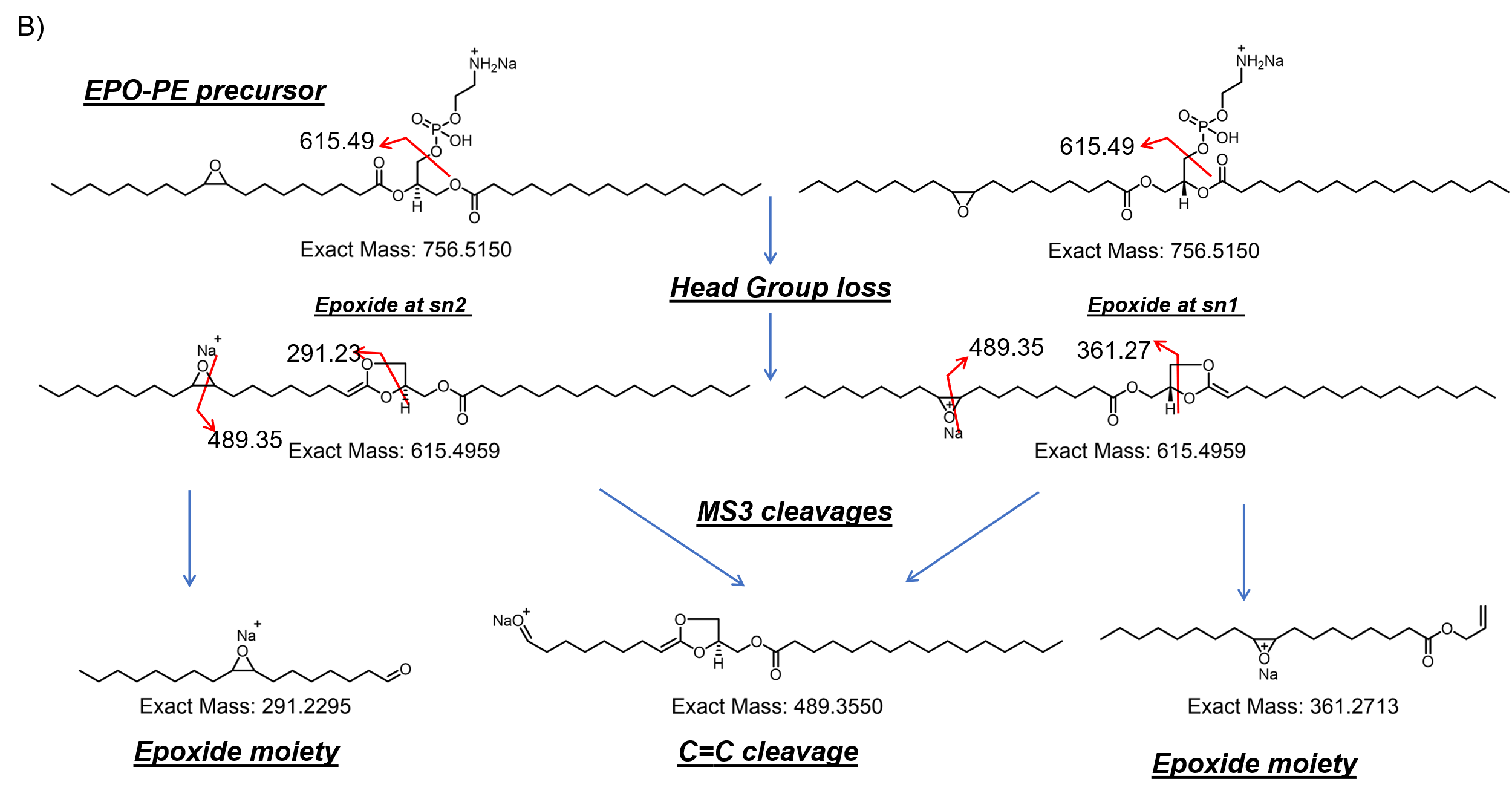


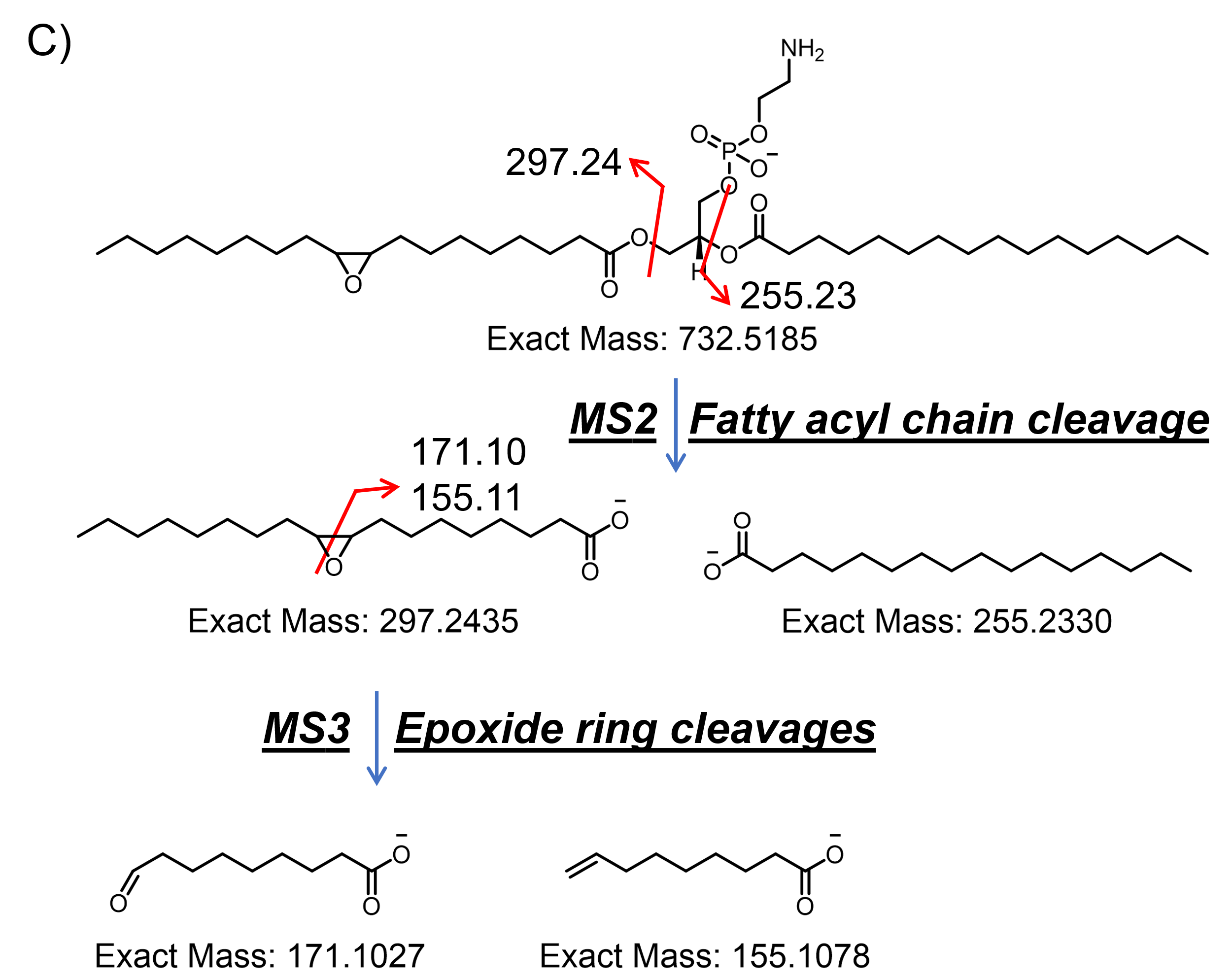


**Figure S18.** Plausible fragments consistent with MS^2^ and MS^3^ of PE 34:0;9Ep: A) Plausible MS^2^ fragments of protonated PE 16:0/18:0;9Ep(cis); B) plausible MS^3^ fragments of PE 16:0/18:0;9Ep(cis) and PE 18:0;9Ep(cis)/16:0 sodium adducts; C) plausible MS^3^ fragments for deprotonated PE 16:0/18:0;9Ep(cis).

**
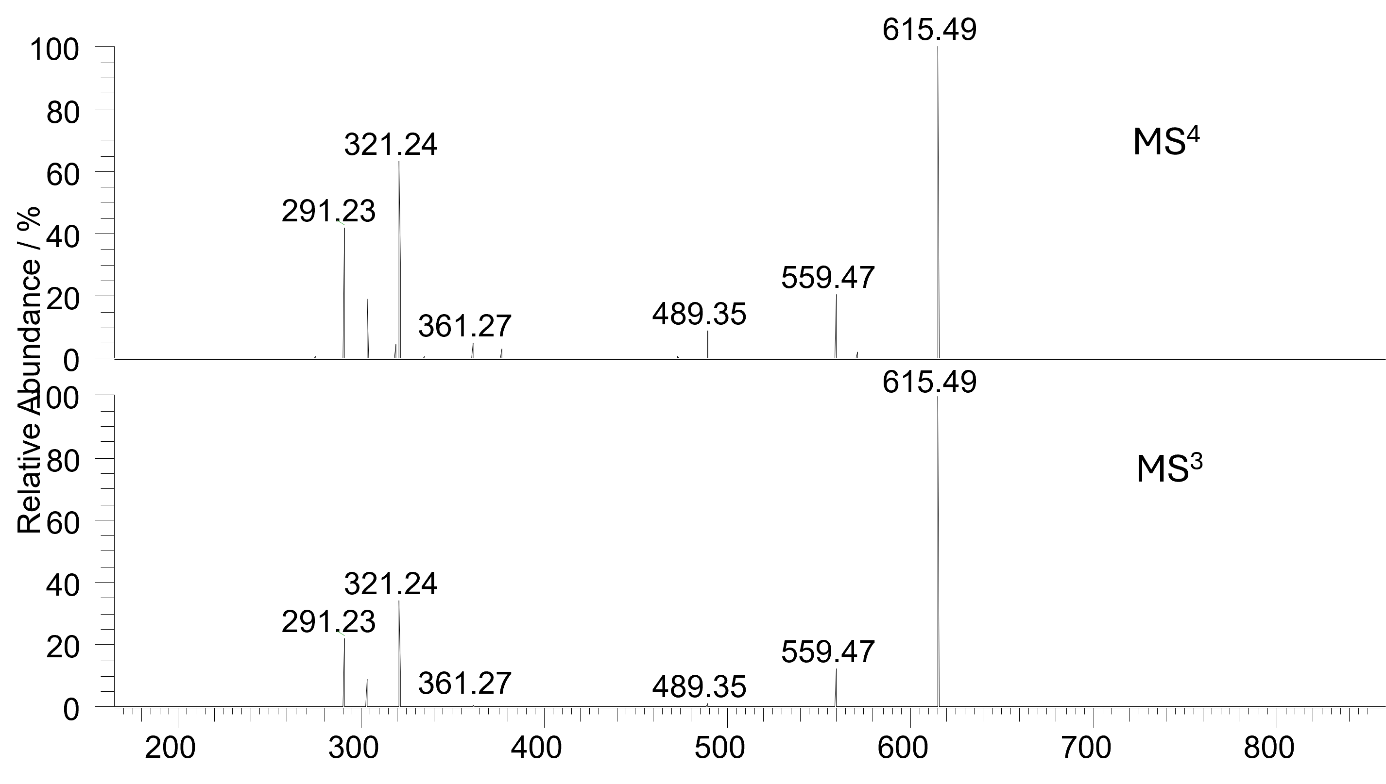
 Figure S19.** Comparison of MS^3^ (from 798 to 615; from 615 to fragments) and MS^4^ (from 798 to 739; from 739 to 615; from 615 to fragments) for sodiated epoxidized PC 16:0/18:1(9Z).


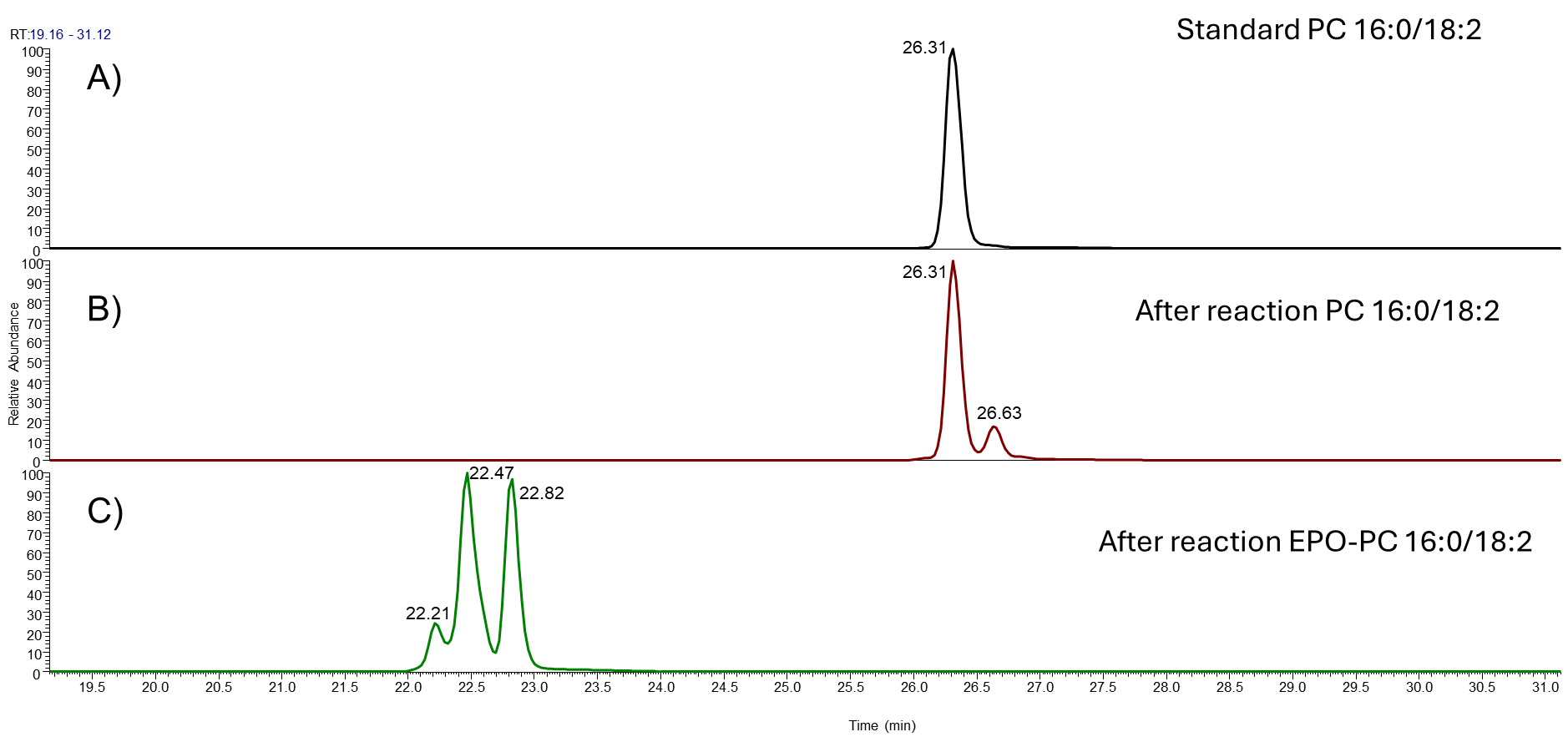


**Figure S20.** [M+H]^+^ trace of the epoxidized product and the pre-/post-reaction reactant profile of PC 16:0/18:2(9Z,12Z). A) EIC profile of the PC 16:0/18:2(9Z,12Z) authentic standard before reaction. B) EIC profile of the PC 16:0/18:2(9Z,12Z) authentic standard after reaction C) Extracted ion chromatogram (EIC) of the mono-epoxidized product.


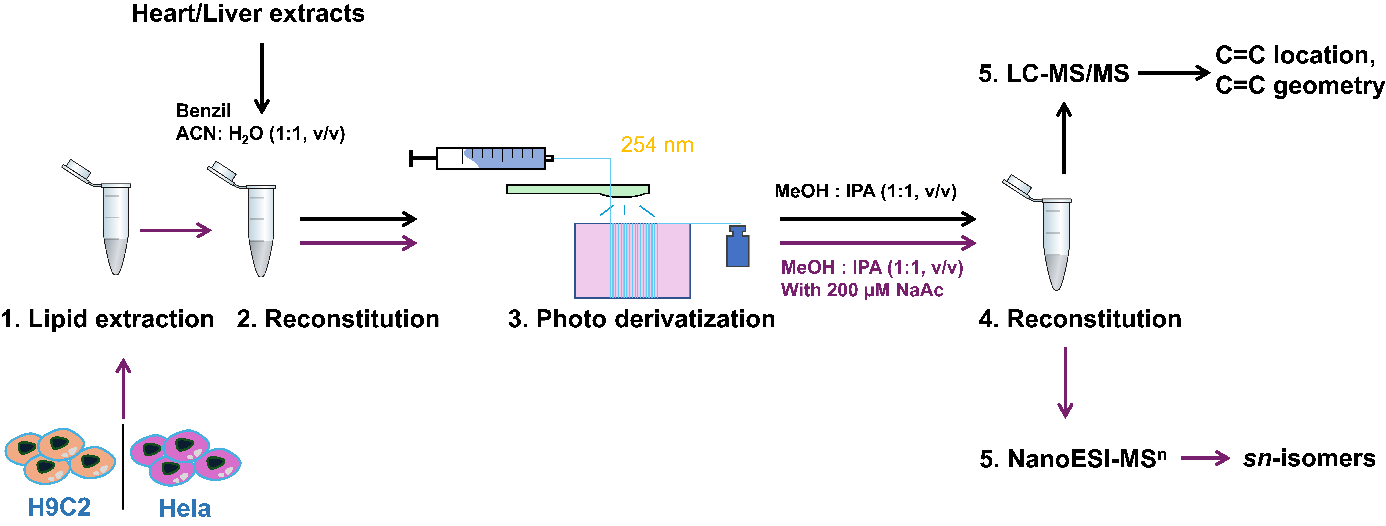


**Scheme S1**: Schematic of the benzil-mediated photo-epoxidation of unsaturated lipids and its practical application for biological samples lipid extracts in this paper for C=C pinpointing, C=C geometry and sn-positions measurements.

| 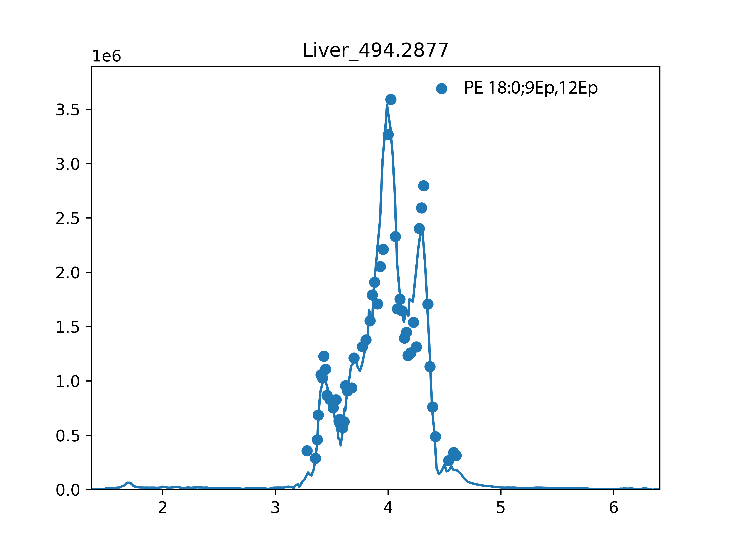 | 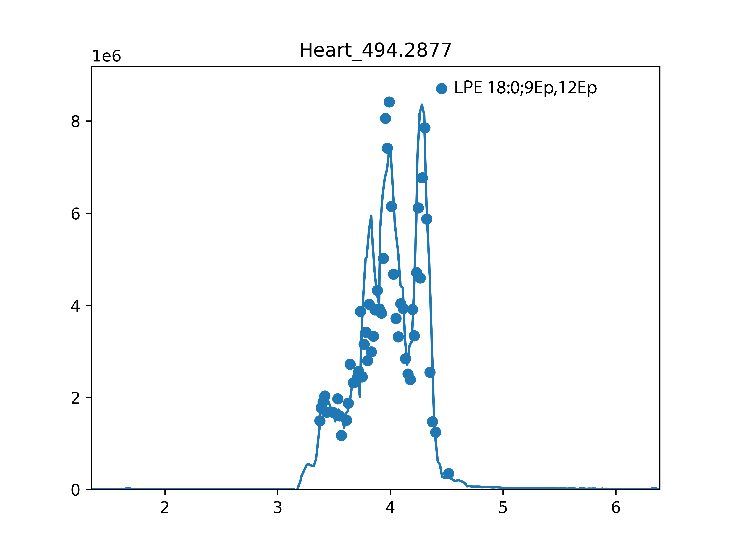 |
| --- | --- |
| 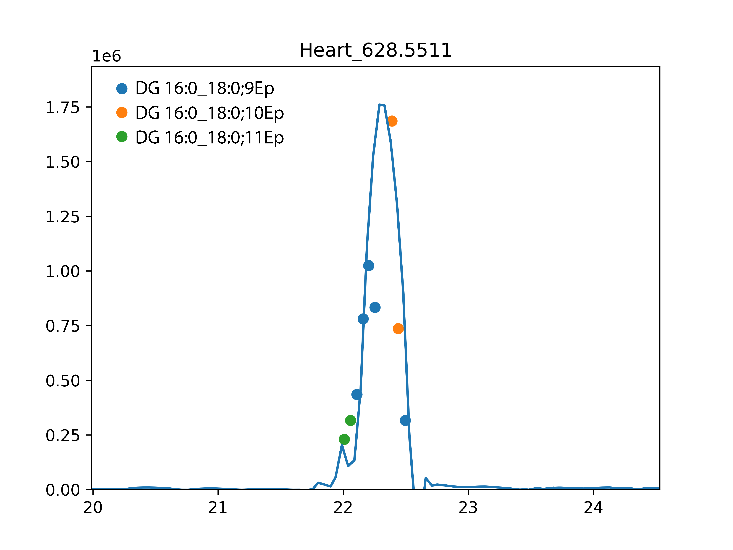 | 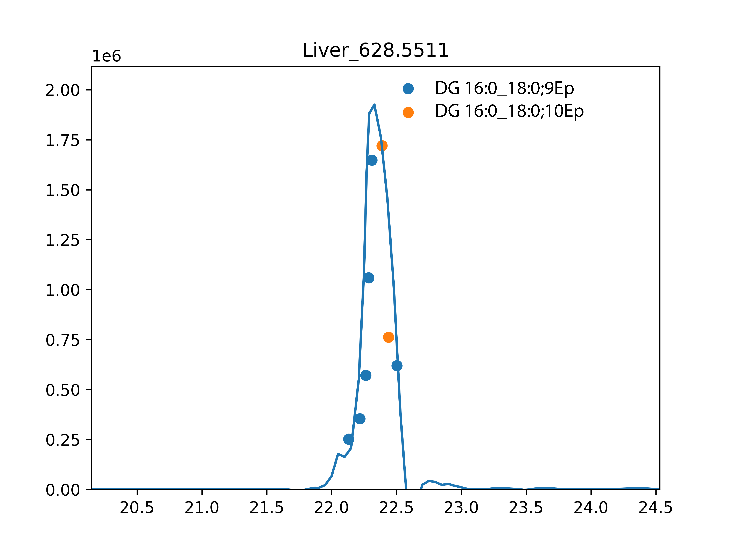 |
| 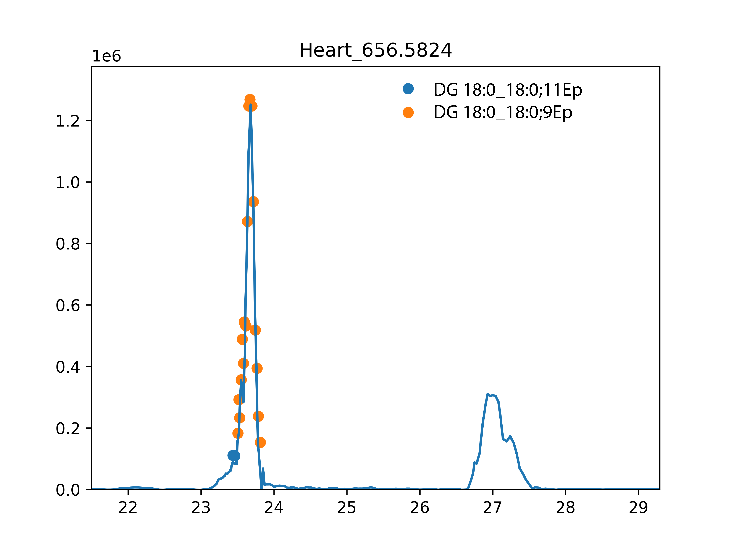 | 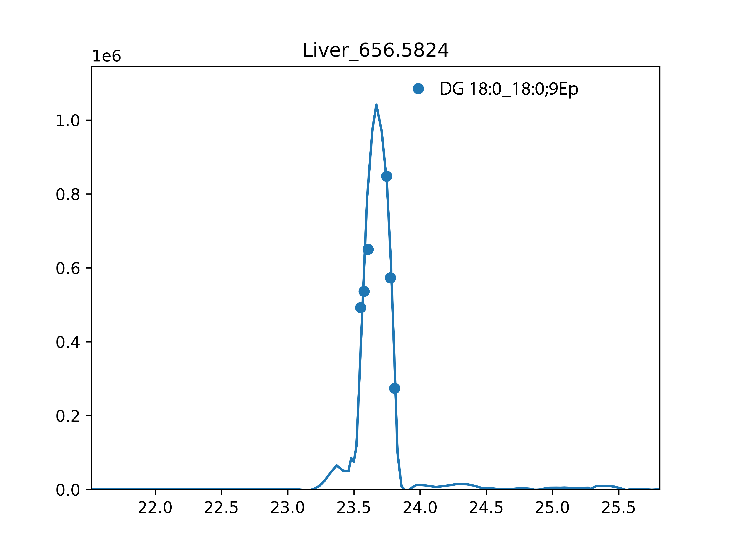 |
| 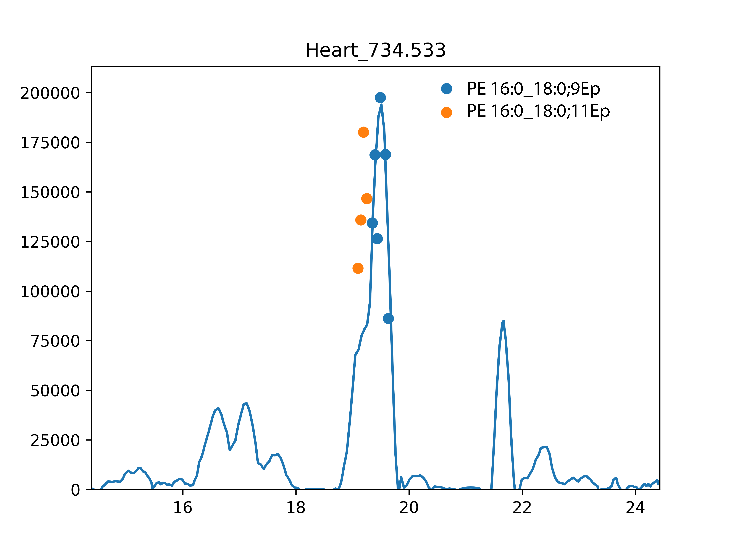 | 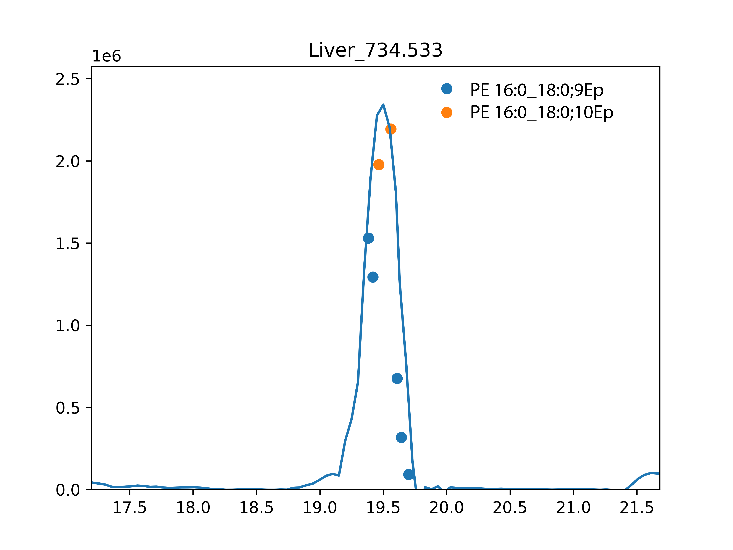 |
| 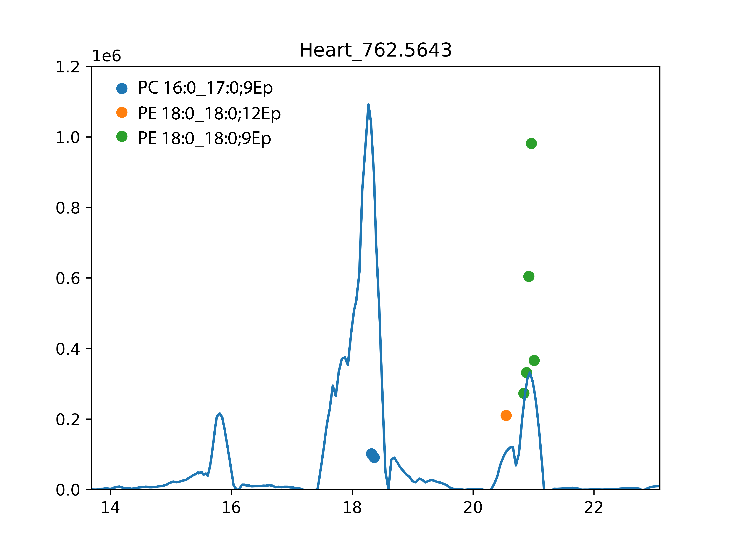 | 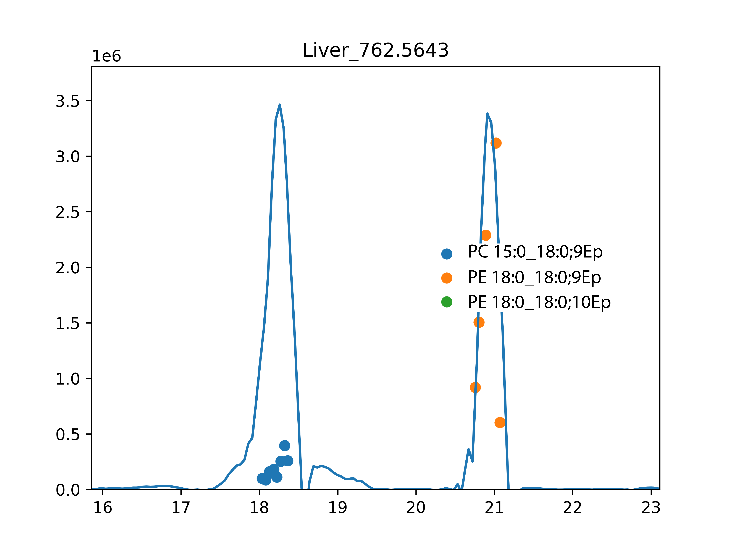 |
| 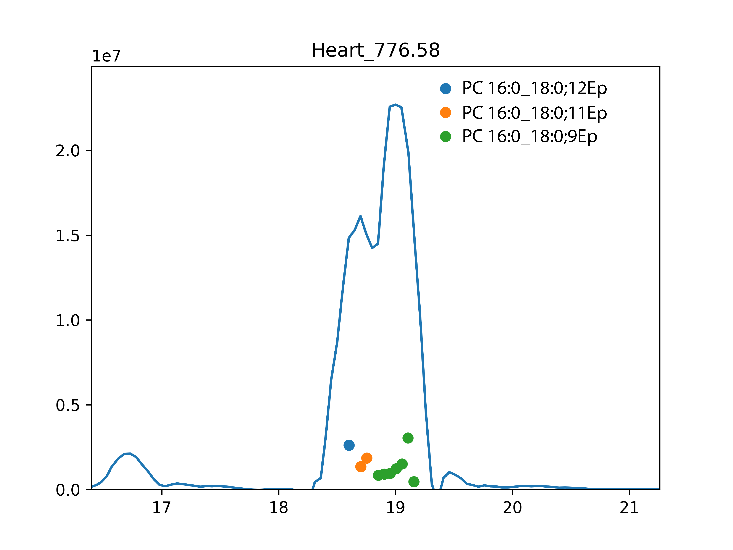 | 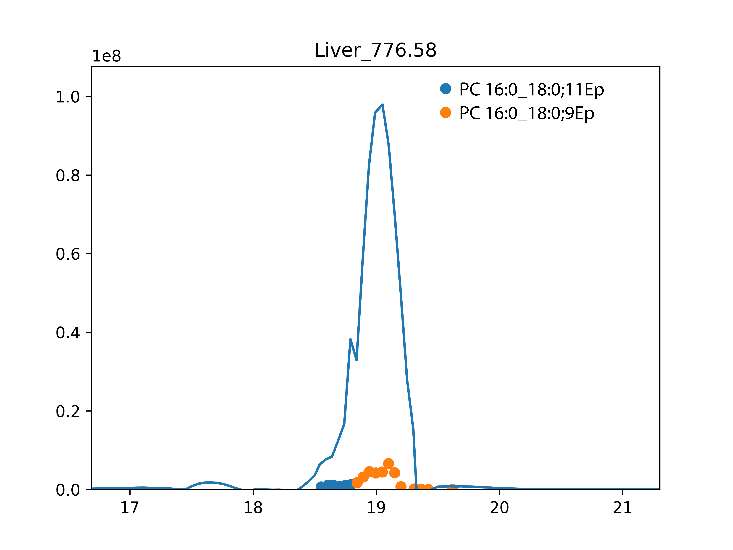 |
| 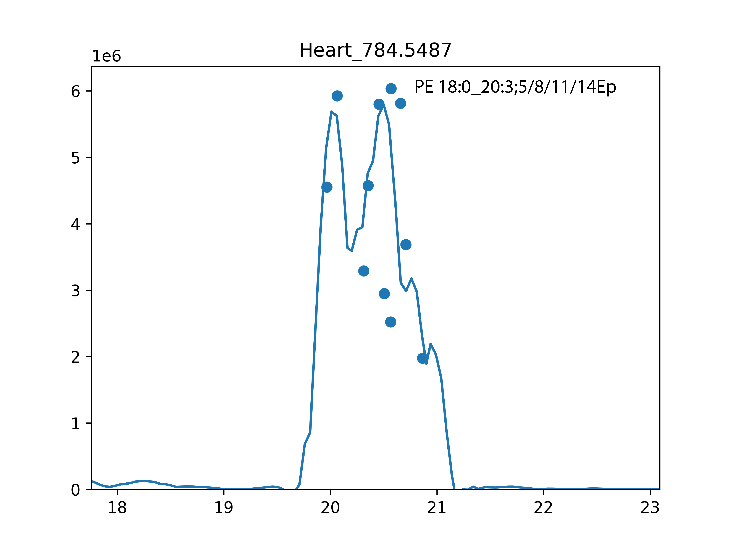 | 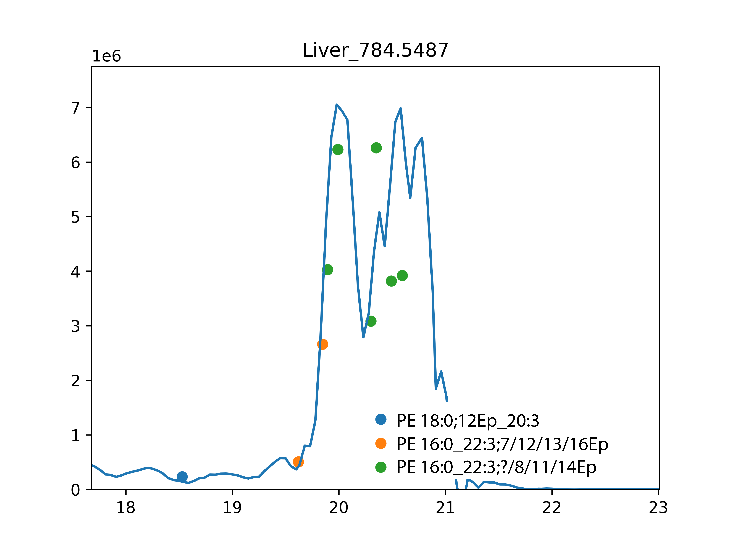 |
| 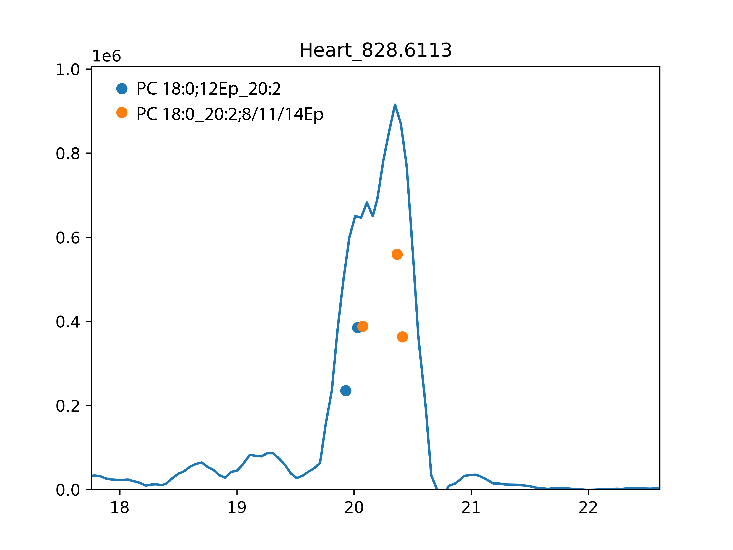 | 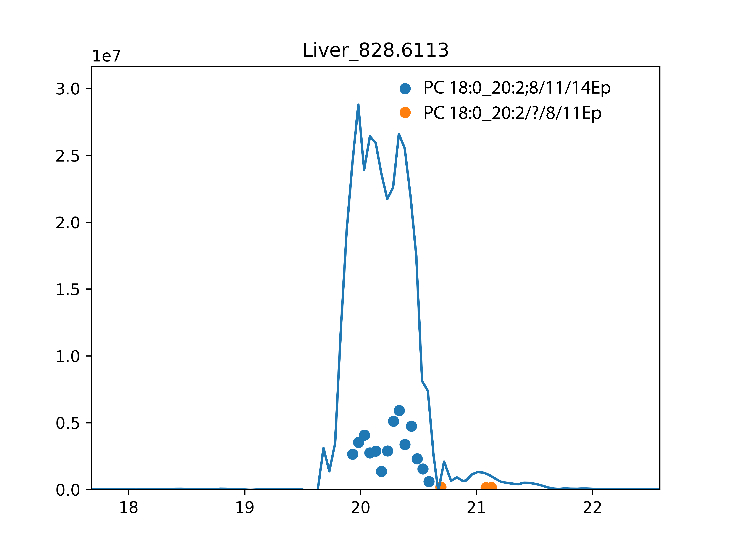 |
| 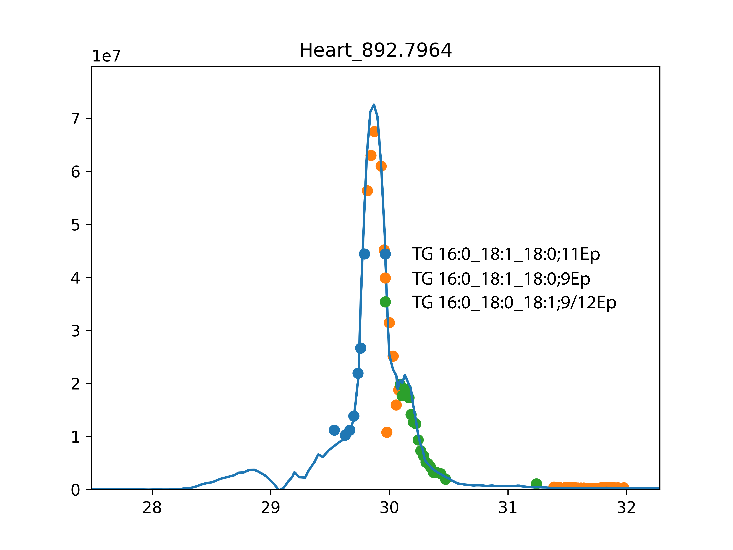 | 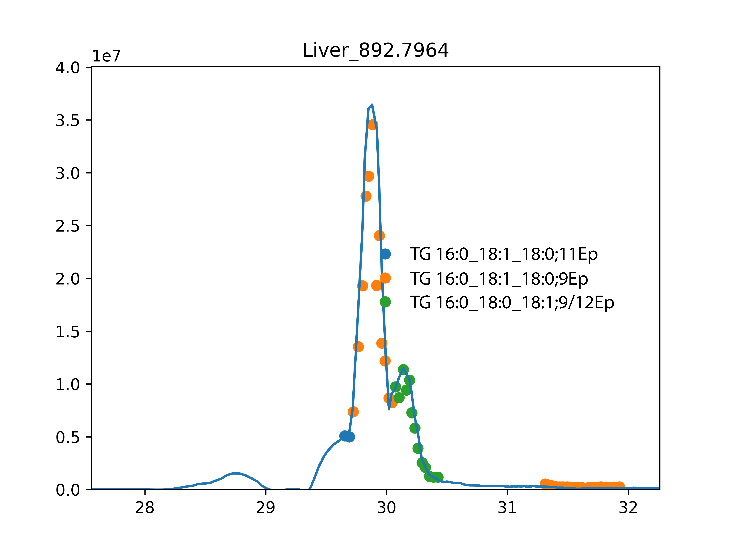 |

**Figure S21.** EIC of some lipids in heart and liver extracts after epoxidation. Identified lipids are indicated and time stamps MS^2^ spectra and associated annotations of these MS^2^ are color coded accordingly.

**Table S5.** Lipid isomers in bovine heart extract and identified corresponding diagnostic fragments ions of heart and liver extract LC-MS^2^ results. Red color highlights indicate missing fragments, black indicates detected fragments, and green highlights detected DB position diagnostic fragments.

| Lipids | Ep-m/z | Double bond location | Characteristic ions | Annotations |
| --- | --- | --- | --- | --- |
| LPE 18:2 | 494.2877 | LPE 18:2(Δ9,Δ12) | 494.2877, 476.2772, 458.2666, 433.235, 353.2686, 335.2581, **370.1625, 410.1938, 354.1676, 394.1989** | PI, PI-water, PI-water2, PI-amino, PI-Headgroup, PI-Headgroup-water, **DB_9, DB_12, DB_9_O, DB_12_O** |
| LPE 20:4 | 518.2877 | LPE 20:4(Δ5,Δ8,Δ11,Δ14) | 518.2877, 500.2772, 482.2666, 457.235, 377.2686, 359.2581, **314.0999, 354.1312, 394.1625, 434.1938, 298.105,** **338.1363, 378.1676, 418.1989** | PI, PI-water, PI-water2, PI-amino, PI-Headgroup, PI-Headgroup-water, **DB_5, DB_8, DB_11, DB_14,** **DB_5_O,** **DB_8_O, DB_11_O, DB_14_O** |
| LPC 18:2 | 536.3347 | LPC 18:2 (Δ9,Δ12) | 536.3347, 518.3241, 500.3136, 184.0733, **412.2095, 452.2408, 396.2146, 436.2459** | PI, PI-water, PI-water2, Headgroup, **DB_9, DB_12, DB_9_O, DB_12_O** |
| LPC 18:1 | 538.3503 | LPC 18:1(Δ12) | 538.3503, 520.3398, 502.3292, 184.0733, **454.2564, 438.2615** | PI, PI-water, PI-water2, Headgroup, **DB_12, DB_12_O** |
|  |  | LPC 18:1(Δ11) | 538.3503, 520.3398, 502.3292, 184.0733, **440.2408, 424.2459** | PI, PI-water, PI-water2, Headgroup, **DB_11, DB_11_O** |
|  |  | LPC 18:1(Δ9) | 538.3503, 520.3398, 502.3292, 184.0733, **412.2095, 396.2146** | PI, PI-water, PI-water2, Headgroup, **DB_9, DB_9_O** |
| LPC 20:4 | 560.3347 | LPC 20:4(Δ5,Δ8,Δ11,Δ14) | 560.3347, 542.3241, 524.3136, 184.0733, **356.1469, 396.1782, 436.2095, 476.2408, 340.152, 380.1833, 420.2146, 460.2459** | PI, PI-water, PI-water2, Headgroup, **DB_5, DB_8, DB_11, DB_14, DB_5_O, DB_8_O, DB_11_O, DB_14_O** |
| LPC 20:3 | 562.3503 | LPC 20:3(Δ8,Δ11,Δ14) | 562.3503, 544.3398, 526.3292, 184.0733, **398.1938, 438.2251, 478.2564, 382.1989, 422.2302, 462.2615** | PI, PI-water, PI-water2, Headgroup, **DB_8, DB_11, DB_14, DB_8_O, DB_11_O, DB_14_O** |
| LPC 22:4 | 588.3660 | LPC 22:4(Δ7, Δ10, Δ13*, Δ16) | 588.366, 570.3554, 552.3449, 184.0733, **384.1782, 424.2095, 464.2408, 504.2721, 368.1833, 408.2146, 448.2459, 488.2772** | PI, PI-water, PI-water2, Headgroup, **DB_7, DB_10, DB_13, DB_16, DB_7_O, DB_10_O, DB_13_O, DB_16_O** |
| DG 34:1 | 628.5511 | DG 18:1(Δ11)_16:0 | 628.5511, 313.2737, 355.2843, **495.4044, 479.4095** | PI, PI-FA1, PI-FA2, **Sn1_DB_11, Sn1_DB_11_O** |
|  |  | DG 18:1(Δ10)_16:0 | 628.5511, 313.2737, 355.2843, **481.3888, 465.3938** | PI, PI-FA1, PI-FA2, **Sn1_DB_10, Sn1_DB_10_O** |
|  |  | DG 18:1(Δ9)_16:0 | 628.5511, 313.2737, 355.2843, **467.3731, 451.3782** | PI, PI-FA1, PI-FA2, **Sn1_DB_9, Sn1_DB_9_O** |
| DG 36:3 | 652.5511 | DG 18:1(Δ9)_18:2 | 652.5511, 337.2737, 355.2843, **491.3731, 475.3782** | PI, PI-FA1, PI-FA2, **Sn1_DB_9, Sn1_DB_9_O** |
|  |  | DG 18:2(Δ9,Δ12)_18:1 | 652.5511, 339.2894, 353.2686, **493.3888, 477.3938, 533.4201, 517.4251** | PI, PI-FA1, PI-FA2, **Sn1_DB_9, Sn1_DB_9_O, Sn1_DB_12**, **Sn1_DB_12_O** |
| DG 36:2 | 654.5667 | DG 18:1(Δ12)_18:1 | 654.5667, 339.2894, 355.2843, **535.4357,** **519.4408** | PI, PI-FA1, PI-FA2, Sn1_DB_12, **Sn1_DB_12_O** |
|  |  | DG 18:1(Δ11)_18:1 | 654.5667, 339.2894, 355.2843, **521.4201,** **505.4251** | PI, PI-FA1, PI-FA2, **Sn1_DB_11, Sn1_DB_11_O** |
|  |  | DG 18:1(Δ10)_18:1 | 654.5667, 339.2894, 355.2843, **507.4044, 491.4095** | PI, PI-FA1, PI-FA2, **Sn1_DB_10, Sn1_DB_10_O** |
|  |  | DG 18:1(Δ9)_18:1 | 654.5667, 339.2894, 355.2843, **493.3888, 477.3938** | PI, PI-FA1, PI-FA2, **Sn1_DB_9, Sn1_DB_9_O** |
|  |  | DG 18:2(Δ9,Δ12)_18:0 | 654.5667, 341.305, 353.2686, **495.4044, 479.4095,** **535.4357,** **519.4408** | PI, PI-FA1, PI-FA2, **Sn1_DB_9, Sn1_DB_9_O,** **Sn1_DB_12,** **Sn1_DB_12_O** |
| DG 36:1 | 656.5824 | DG 18:1(Δ11)_18:0 | 656.5824, 341.305, 355.2843, **523.4357, 507.4408** | PI, PI-FA1, PI-FA2, **Sn1_DB_11, Sn1_DB_11_O** |
|  |  | DG 18:1(Δ9)_18:0 | 656.5824, 341.305, 355.2843, **495.4044, 479.4095** | PI, PI-FA1, PI-FA2, **Sn1_DB_9, Sn1_DB_9_O** |
| PE 34:2 | 732.5174 | PE 18:2(Δ9,Δ12)_16:0 | 732.5174, 714.5068, 671.4646, 591.4983, 573.4877, 353.2686, 335.2581, 313.2737, **467.3731, 451.3782, 507.4044, 491.4095** | PI, PI-water, PI-amino, PI-HG, PI-HG-water, sn1_FA, sn2_FA, sn1_FA_water, **Sn1_DB_9, Sn1_DB_9_O, Sn1_DB_12, Sn1_DB_12_O** |
| PE 34:1 | 734.5330 | PE 18:1(Δ11)_16:0 | 734.533, 716.5225, 673.4803, 593.514, 575.5034, 355.2843, 337.2737, 313.2737, **495.4044, 479.4095** | PI, PI-water, PI-amino, PI-HG, PI-HG-water, sn1_FA, sn2_FA, sn1_FA_water, **Sn1_DB_11, Sn1_DB_11_O** |
|  |  | PE 18:1(Δ9)_16:0 | 734.533, 716.5225, 673.4803, 593.514, 575.5034, 355.2843, 337.2737, 313.2737, **467.3731, 451.3782** | PI, PI-water, PI-amino, PI-HG, PI-HG-water, sn1_FA, sn2_FA, sn1_FA_water, **Sn1_DB_9, Sn1_DB_9_O** |
| PC 32:1 | 748.5487 | PC 16:1(Δ9)_16:0 | 748.5487, 730.5381, 565.4827, 547.4721, 496.3398, 478.3292, 510.319, 492.3085, **650.4391, 634.4442** | PI, PI-water, PI-HG, PI-HG-water, PI-FA1, PI-FA1-water, PI-FA2, PI-FA2-water, **Sn1_DB_9, Sn1_DB_9_O** |
| PE 36:2 | 760.5487 | PE 18:2(Δ9,Δ12)_18:0 | 760.5487, 742.5381, 699.4959, 619.5296, 601.519, 353.2686, 335.2581, 341.305, **495.4044, 479.4095, 535.4357, 519.4408** | PI, PI-water, PI-amino, PI-HG, PI-HG-water, sn1_FA, sn2_FA, sn1_FA_water, **Sn1_DB_9, Sn1_DB_9_O, Sn1_DB_12, Sn1_DB_12_O** |
| PE 36:1 | 762.5643 | PE 18:1(Δ12)_18:0 | 762.5643, 744.5538, 701.5116, 621.5453, 603.5347, 355.2843, 337.2737, 341.305, **537.4514, 521.4564** | PI, PI-water, PI-amino, PI-HG, PI-HG-water, sn1_FA, sn2_FA, sn1_FA_water, **Sn1_DB_12, Sn1_DB_12_O** |
|  |  | PE 18:1(Δ9)_18:0 | 762.5643, 744.5538, 701.5116, 621.5453, 603.5347, 355.2843, 337.2737, 341.305, **495.4044, 479.4095** | PI, PI-water, PI-amino, PI-HG, PI-HG-water, sn1_FA, sn2_FA, sn1_FA_water, **Sn1_DB_9, Sn1_DB_9_O** |
| PC 33:1 |  | PC 17:1(Δ9)_16:0 | 762.5643, 744.5538, 579.4983, 561.4877, 496.3398, 478.3292, 524.3347, 506.3241, **650.4391, 634.4442** | PI, PI-water, PI-HG, PI-HG-water, PI-FA1, PI-FA1-water, PI-FA2, PI-FA2-water, **Sn1_DB_9, Sn1_DB_9_O** |
| PC 34:3 | 772.5487 | PC 18:3(Δ9, Δ12, Δ15*)_16:0 | 772.5487, 754.5381, 589.4827, 571.4721, 496.3398, 478.3292, 534.319, 516.3085, **650.4391,** **634.4442,** **690.4704, 674.4755,** **730.5017, 714.5068** | PI, PI-water, PI-HG, PI-HG-water, PI-FA1, PI-FA1-water, PI-FA2, PI-FA2-water, **Sn1_DB_9,** **Sn1_DB_9_O,** **Sn1_DB_12, Sn1_DB_12_O,** **Sn1_DB_15, Sn1_DB_15_O** |
| PC 34:2 | 774.5643 | PC 18:2(Δ9,Δ12)_16:0 | 774.5643, 756.5538, 591.4983, 573.4877, 496.3398, 478.3292, 536.3347, 518.3241, **650.4391, 634.4442, 690.4704, 674.4755** | PI, PI-water, PI-HG, PI-HG-water, PI-FA1, PI-FA1-water, PI-FA2, PI-FA2-water, **Sn1_DB_9, Sn1_DB_9_O, Sn1_DB_12, Sn1_DB_12_O** |
| PC 34:1 | 776.5800 | PC 18:1(Δ12)_16:0 | 776.58, 758.5694, 593.514, 575.5034, 496.3398, 478.3292, 538.3503, 520.3398, **692.4861, 676.4912** | PI, PI-water, PI-HG, PI-HG-water, PI-FA1, PI-FA1-water, PI-FA2, PI-FA2-water, **Sn1_DB_12, Sn1_DB_12_O** |
|  |  | PC 18:1(Δ11)_16:0 | 776.58, 758.5694, 593.514, 575.5034, 496.3398, 478.3292, 538.3503, 520.3398, **678.4704, 662.4755** | PI, PI-water, PI-HG, PI-HG-water, PI-FA1, PI-FA1-water, PI-FA2, PI-FA2-water, **Sn1_DB_11, Sn1_DB_11_O** |
|  |  | PC 18:1(Δ9)_16:0 | 776.58, 758.5694, 593.514, 575.5034, 496.3398, 478.3292, 538.3503, 520.3398, **650.4391, 634.4442** | PI, PI-water, PI-HG, PI-HG-water, PI-FA1, PI-FA1-water, PI-FA2, PI-FA2-water, **Sn1_DB_9, Sn1_DB_9_O** |
| PE 38:4 | 784.5487 | PE 20:4(Δ5,Δ8,Δ11,Δ14)_18:0 | 784.5487, 766.5381, 723.4959, 643.5296, 625.519, 377.2686, 359.2581, 341.305, **439.3418, 423.3469, 479.3731, 463.3782, 519.4044, 503.4095, 559.4357, 543.4408** | PI, PI-water, PI-amino, PI-HG, PI-HG-water, sn1_FA, sn2_FA, sn1_FA_water, **Sn1_DB_5, Sn1_DB_5_O, Sn1_DB_8, Sn1_DB_8_O, Sn1_DB_11, Sn1_DB_11_O, Sn1_DB_14, Sn1_DB_14_O** |
| PC 36:3 | 800.5800 | PC 20:3(Δ8,Δ11,Δ14)_16:0 | 800.58, 782.5694, 617.514, 599.5034, 496.3398, 478.3292, 562.3503, 544.3398, **636.4235, 620.4286, 676.4548, 660.4599, 716.4861, 700.4912** | PI, PI-water, PI-HG, PI-HG-water, PI-FA1, PI-FA1-water, PI-FA2, PI-FA2-water, **Sn1_DB_8, Sn1_DB_8_O, Sn1_DB_11, Sn1_DB_11_O, Sn1_DB_14, Sn1_DB_14_O** |
|  |  | PC 18:2(Δ9,Δ12)_18:1 | 800.58, 782.5694, 617.514, 599.5034, 522.3554, 504.3449, 536.3347, 518.3241, **676.4548, 660.4599, 716.4861, 700.4912** | PI, PI-water, PI-HG, PI-HG-water, PI-FA1, PI-FA1-water, PI-FA2, PI-FA2-water, **Sn1_DB_9, Sn1_DB_9_O, Sn1_DB_12, Sn1_DB_12_O** |
| PC 36:2 | 802.5956 | PC 18:1(Δ12)_18:1 | 802.5956, 784.5851, 619.5296, 601.519, 522.3554, 504.3449, 538.3503, 520.3398, **718.5017, 702.5068** | PI, PI-water, PI-HG, PI-HG-water, PI-FA1, PI-FA1-water, PI-FA2, PI-FA2-water, **Sn1_DB_12, Sn1_DB_12_O** |
|  |  | PC 18:1(Δ9)_18:1 | 802.5956, 784.5851, 619.5296, 601.519, 522.3554, 504.3449, 538.3503, 520.3398, **676.4548, 660.4599** | PI, PI-water, PI-HG, PI-HG-water, PI-FA1, PI-FA1-water, PI-FA2, PI-FA2-water, **Sn1_DB_9, Sn1_DB_9_O** |
|  |  | PC 18:2(Δ9,Δ12)_18:0 | 802.5956, 784.5851, 619.5296, 601.519, 524.3711, 506.3605, 536.3347, 518.3241, **678.4704, 662.4755, 718.5017, 702.5068** | PI, PI-water, PI-HG, PI-HG-water, PI-FA1, PI-FA1-water, PI-FA2, PI-FA2-water, **Sn1_DB_9, Sn1_DB_9_O, Sn1_DB_12, Sn1_DB_12_O** |
| PS 36:1 | 806.5542 | PS 18:1(Δ12)_18:0 | 806.5542, 788.5436, 698.4881, 621.5453, 603.5347, 355.2843, 337.2737, 341.305, **537.4514, 521.4564** | PI, PI-water, PI-amino, PI-HG, PI-HG-water, sn1-FA, sn2-FA, sn1_FA_water, **Sn1_DB_12, Sn1_DB_12_O** |
|  |  | PS 18:1(Δ9)_18:0 | 806.5542, 788.5436, 698.4881, 621.5453, 603.5347, 355.2843, 337.2737, 341.305, **495.4044, 479.4095** | PI, PI-water, PI-amino, PI-HG, PI-HG-water, sn1-FA, sn2-FA, sn1_FA_water, **Sn1_DB_9, Sn1_DB_9_O** |
| TG 52:2 | 892.7964 | TG 18:1(Δ11)_18:1_16:0 | 892.7964, 577.519, 593.514, 619.5296, **495.4044, 521.4201, 479.4095, 505.4251** | PI, PI-FA1, PI-FA2, PI-FA3, **Sn1_DB_11Loss_Sn2, Sn1_DB_11Loss_Sn3, Sn1_DB_11Loss_Sn2_O, Sn1_DB_11Loss_Sn3_O** |
|  |  | TG 18:1(Δ9)_18:1_16:0 | 892.7964, 577.519, 593.514, 619.5296, **467.3731, 493.3888, 451.3782, 477.3938** | PI, PI-FA1, PI-FA2, PI-FA3, **Sn1_DB_9Loss_Sn2, Sn1_DB_9Loss_Sn3, Sn1_DB_9Loss_Sn2_O, Sn1_DB_9Loss_Sn3_O** |
|  |  | TG 18:2(Δ9,Δ12)_18:0_16:0 | 892.7964, 579.5347, 591.4983, 619.5296, **467.3731, 495.4044, 451.3782, 479.4095, 507.4044, 535.4357, 491.4095, 519.4408** | PI, PI-FA1, PI-FA2, PI-FA3, **Sn1_DB_9Loss_Sn2, Sn1_DB_9Loss_Sn3, Sn1_DB_9Loss_Sn2_O, Sn1_DB_9Loss, Sn1_DB_12Loss_Sn2, Sn1_DB_12Loss_Sn3, Sn1_DB_12Loss_Sn2_O, Sn1_DB_12Loss_Sn3_O** |

| 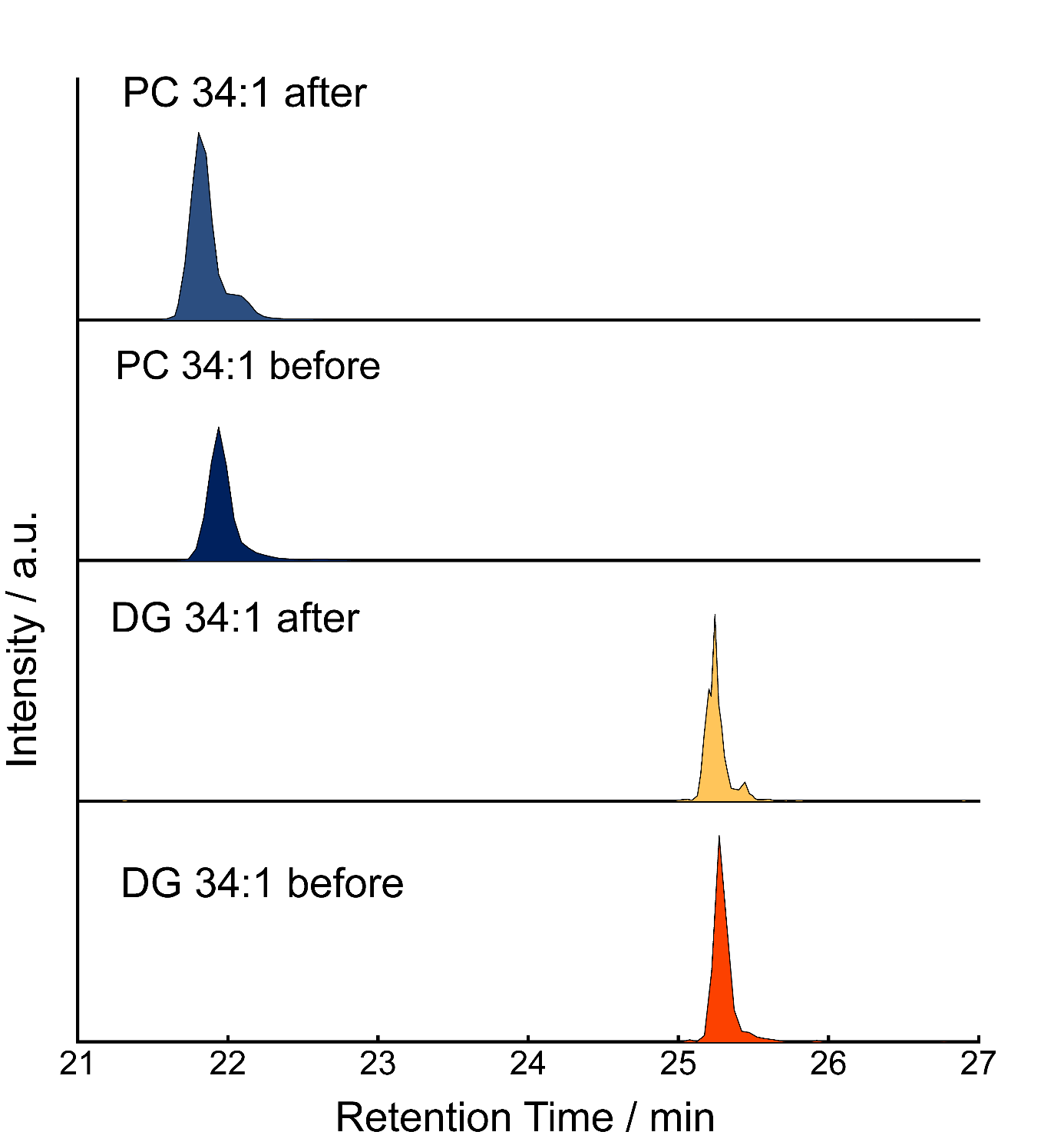 |  |
| --- | --- |

**Figure S22.** EIC of selected GPLs with mono-unsaturated FAs before and after the photochemical epoxidation with benzil in liver extract.

**References**

1. Feng, Y.; Chen, B.; Yu, Q.; Li, L., Identification of Double Bond Position Isomers in Unsaturated Lipids by m-CPBA Epoxidation and Mass Spectrometry Fragmentation. *Anal. Chem.* **2019,** *91* (3), 1791-1795 <https://doi.org/10.1021/acs.analchem.8b04905>.

2. Huang, L.; Huang, M.; Zhou, T., Efficient Strategy for Characterization and Quantification of Polyunsaturated Lipids by Microwave-Assisted MMPP Epoxidation. *Anal. Chem.* **2024,** *96* (28), 11189-11197 <https://doi.org/10.1021/acs.analchem.4c00410>.

3. Cao, W.; Cheng, S.; Yang, J.; Feng, J.; Zhang, W.; Li, Z.; Chen, Q.; Xia, Y.; Ouyang, Z.; Ma, X., Large-scale lipid analysis with C=C location and sn-position isomer resolving power. *Nat. Commun.* **2020,** *11* (1), 375 <https://doi.org/10.1038/s41467-019-14180-4>.

4. Becher, S.; Berden, G.; Martens, J.; Oomens, J.; Heiles, S., IRMPD Spectroscopy of [PC (4:0/4:0) + M]+ (M = H, Na, K) and Corresponding CID Fragment Ions. *J. Am. Soc. Mass Spectrom.* **2021,** *32* (12), 2874-2884 <https://doi.org/10.1021/jasms.1c00277>.

5. Kirschbaum, C.; Greis, K.; Polewski, L.; Gewinner, S.; Schöllkopf, W.; Meijer, G.; von Helden, G.; Pagel, K., Unveiling Glycerolipid Fragmentation by Cryogenic Infrared Spectroscopy. *J. Am. Chem. Soc.* **2021,** *143* (36), 14827-14834 <https://doi.org/10.1021/jacs.1c06944>.
